# Evolutionary history of phosphatidylinositol- 3-kinases: ancestral origin in eukaryotes and complex duplication patterns

**DOI:** 10.1186/s12862-015-0498-7

**Published:** 2015-10-19

**Authors:** Héloïse Philippon, Céline Brochier-Armanet, Guy Perrière

**Affiliations:** Laboratoire de Biométrie et Biologie Evolutive, UMR CNRS 5558, Université Claude Bernard – Lyon 1, 43 bd. du 11 Novembre 1918, Villeurbanne, 69622 France

**Keywords:** Phosphatidylinositol-3-kinases, phylogeny, signalling pathway, LECA

## Abstract

**Background:**

Phosphatidylinositol-3-kinases (PI3Ks) are a family of eukaryotic enzymes modifying phosphoinositides in phosphatidylinositols-3-phosphate. Located upstream of the AKT/mTOR signalling pathway, PI3Ks activate secondary messengers of extracellular signals. They are involved in many critical cellular processes such as cell survival, angiogenesis and autophagy. PI3K family is divided into three classes, including 14 human homologs. While class II enzymes are composed of a single catalytic subunit, class I and III also contain regulatory subunits. Here we present an in-depth phylogenetic analysis of all PI3K proteins.

**Results:**

We confirmed that PI3K catalytic subunits form a monophyletic group, whereas regulatory subunits form three distinct groups. The phylogeny of the catalytic subunits indicates that they underwent two major duplications during their evolutionary history: the most ancient arose in the Last Eukaryotic Common Ancestor (LECA) and led to the emergence of class III and class I/II, while the second – that led to the separation between class I and II – occurred later, in the ancestor of Unikonta (*i.e.*, the clade grouping Amoebozoa, Fungi, and Metazoa). These two major events were followed by many lineage specific duplications in particular in vertebrates, but also in various protist lineages. Major loss events were also detected in Vidiriplantae and Fungi. For the regulatory subunits, we identified homologs of class III in all eukaryotic groups indicating that, for this class, both the catalytic and the regulatory subunits were presents in LECA. In contrast, homologs of the regulatory class I have a more recent origin.

**Conclusions:**

The phylogenetic analysis of the PI3K shed a new light on the evolutionary history of these enzymes. We found that LECA already contained a PI3K class III composed of a catalytic and a regulatory subunit. Absence of class II regulatory subunits and the recent origin of class I regulatory subunits is puzzling given that the class I/II catalytic subunit was present in LECA and has been conserved in most present-day eukaryotic lineages. We also found surprising major loss and duplication events in various eukaryotic lineages. Given the functional specificity of PI3K proteins, this suggests dynamic adaptation during the diversification of eukaryotes.

**Electronic supplementary material:**

The online version of this article (doi:10.1186/s12862-015-0498-7) contains supplementary material, which is available to authorized users.

## Background

Phosphatidylinositol-3-kinases (PI3Ks) are enzymes that phosphorylate the 3’-position of inositol ring to generate different phosphoinositides (PIs). They are involved in many critical cellular processes such as cell survival, angiogenesis [1] or autophagy[2] and are deregulated in many human disorders (see below). PI3Ks were discovered in the 1980’s as a consequence of the growing interest for their products. Following their identification and first cDNA clones, their two main inhibitors, Wortmannin and LY294002, were discovered in 1993 and 1994 respectively [3, 4]. Domain organisation of PI3Ks was already partially discovered in 1997 [5] and the first three-dimensional protein structure was resolved two years later [6] (see [7] for a detailed review on the discovery of PI3Ks). They are divided into three classes depending on their substrate (I, II and III), and 14 coding genes have been identified in human (see Table 1 for a complete nomenclature).

**Table 1:** Nomenclature of the 14 human PI3K proteins

	Common name	Gene name	Ensembl ID	UniProt ID	Length
Catalytic subunits					
IA	p110 *α*	PIK3CA	ENSP00000263967	P42336	1068
	p110 *β*	PIK3CB	ENSP00000418143	P42338	1070
	p110 *δ*	PIK3CD	ENSP00000446444	O00329	1044
IB	p110 *γ*	PIK3CG	ENSP00000392258	P48736	1102
II	PI3K-C2 *α*	PIK3C2A	ENSP00000265970	O00443	1086
	PI3K-C2 *β*	PIK3C2B	ENSP00000356155	O00750	1634
	PI3K-C2 *γ*	PIK3C2G	ENSP00000266497	O75747	1445
III	VPS34	PIK3C3	ENSP00000262039	Q8NEB9	887
Regulatory subunits					
IA	p85 *α*	PIK3R1	ENSP00000274335	P27986	724
	p85 *β*	PIK3R2	ENSP00000222254	O00459	728
	p55 *γ*	PIK3R3	ENSP00000361075	Q92569	461
IB	p101	PIK3R5	ENSP00000392812	Q8WYR1	880
	p87	PIK3R6	ENSP00000475670	Q5UE93	754
III	VPS15	PIK3R4	ENSP00000349205	Q99570	B3

Class I proteins transform phosphatidylinositols-4,5-bisphosphate (PI(4,5)P_2_) into phosphatidylinositols-3,4,5-triphosphate (PI(3,4,5)P_3_). The reverse reaction is done by PTEN (Phosphatase and Tensin homolog), a well known tumour suppressor protein [8, 9]. Class I is subdivided into two groups called IA and IB, depending on whether or not they can bind p85-type regulatory proteins. In human, class IA catalytic subunits (p110 *α*, p110 *β* and p110 *δ*) can bind the p85 *α* (and its two alternatives forms p55 *α* and p50 *α*), p85 *β* and p55 *γ* regulatory subunits. In contrast, p110 *γ*, the only catalytic human protein of class IB, can bind two regulatory subunits named p87 and p101. Class I is the most studied and its members are involved in a lot of human disorders like cancers. For instance, p110 *α* expression is deregulated in more than 30 % of various solid tumours [10], and the corresponding gene is mutated in 25 % of breast tumour samples [11–13], in 15-20 % of colorectal cancers [14–17] and in 10 % of oesophagealgeal cancers [10, 18]. Proteins p110 *α* and p110 *β* are generally ubiquitously expressed, and no major difference in their functions have been discovered. The major activators of class IA are RTKs (Receptor Tyrosine Kinases) [19–21] and IGF1 (Insulin-like Growth Factor 1) [21], whereas class IB is principally activated by GPCRs (G Protein-Coupled Receptors) [19, 21].

Class II proteins (PI3K-C2 *α*, PI3K-C2 *β* and PI3K-C2 *γ*) are the only ones without a regulatory subunit in human, and are the most poorly characterized. Their preferential phosphoinositide substrate is not yet clearly defined and can differ between *in vivo* and *in vitro* studies [22]. In terms of biological impact, it was proved in mouse that PI3K-C2 *α* deficiency results in embryonic lethality caused by defects in vasculogenesis [23, 24]. Another study demonstrates a role in tumour angiogenesis in the context of Lewis lung carcinoma [23]. Activators of class II are chemokines like MCP-1[25], cytokines (TNF- *α* and leptin) [26] and Lysophosphatidic Acid (LPA) [27]. On the contrary, Tamoxifene seems to reduce its expression in mice [23].

Finally, class III proteins synthesize phosphatidylinositols-3-phosphate (PI(3)P) from phosphatidylinositide (PI). This class is made of one catalytic and one regulatory subunits named Vacuolar Protein Sorting 34 (VPS34 or PIK3C3) and Vacuolar Protein Sorting 15 (VPS15), respectively [28]. The role of class III PI3K is to regulate membrane trafficking [28] and autophagosome formation in human [29–31].

While PI3K proteins are well studied in human, little is known about these enzymes in other organisms. Homologs of class III have been reported in unicellular eukaryotes (*e.g.*, *Saccharomyces cerevisiae*, *Schizosaccharomyces pombe*, *Candida albicans*, *Dictyostelium discoideum*), vertebrates, plants, *Caenorhabditis elegans*, *Drosophila melanogaster* [32] and in microalgae [33]. The yeast genome does not code for other classes of PI3K [34]. For the classes I and II, homologs were found in vertebrates, worm, fly and Amoebozoa but not in yeast [28]. From a functional point of view, little information is available in non-human organisms. For Excavata and SAR (Stramenopiles, Alveolata and Rhizaria [35]), studies generally focus on the pathogen impact on the host cell phosphatidylinositols quantity more than on the function of PI3K homologs [36, 37]. Nevertheless, it has been shown that in the apicomplexan *Toxoplasma gondii*, PI3Ks are involved in the shape and size of the apicoplast [38]. In the amoebozoan species *Dictyostelium*, the PI3K classes I and II are activated by GPCRs and are involved in chemotaxis [39–41]. In *Drosophila*, focus has been on EGRF-RAS or EGFR-TOR proteins [42–44], when PTEN deregulation was largely studied in yeast [45]. Finally, a recent study presents the impact of IGFR, a PI3K activator, on the arsenite-induced apoptosis in *C. elegans* [46].

Understanding the evolutionary history of PI3Ks can provide new information about their diversity and functions. More precisely, integrating functional information available from different species and phylogenetic history allows making predictions on the ancestral as well as present day protein functions [47, 48]. Despite their biological interest, only two incomplete phylogenies of PI3Ks have been published to date. The first one was published in 2003 by Kawashima *et al.* [49] and concerned the PI3K catalytic subunits and the class IA, III but not IB regulatory subunits in Opisthokonta species. The second, published in 2011 by Brown and Auger [50], focused on the catalytic subunits in eukaryotes. Both studies identified an ancient gene duplication event that lead to the separation of class III and I/II catalytic subunits that was followed by a more recent duplication at the origin of class I and II. They found homologs of class III catalytic subunit (VPS34) in all eukaryotic groups. Furthermore, the pattern of gene duplications in catalytic class II subunits was consistent between the two studies, but not the one of class I. Therefore, the evolutionary history of PI3K portrayed by those two studies is only partial. Especially, nothing is known about the evolutionary history of class IB regulatory subunit and the existence of non-Opisthokonta homologs of class IA regulatory protein.

Taking the opportunity of the rainfall of genomic data, we performed an in-depth phylogenetic analysis of the PI3K family. First we found that catalytic and regulatory class III proteins were already present in the Last Eukaryotic Common Ancestor (LECA). We inferred that the class I and class II catalytic subunits diverged from the ancestor of Unikonta, and we deciphered the pattern of duplications within classes I and II. We showed that class IA and IB regulatory proteins are of relative recent origin and emerge in the common ancestor shared by Metazoa, Ichthyosporea and Choanoflagellida (MIC) and in the Vertebrata lineage, respectively. Finally, the investigation of the domain composition of PI3K homologs allowed testing some hypotheses resulting from our phylogenetic analysis and provided information on protein functions.

## Material and methods

The 14 human PI3K protein sequences were retrieved from UniProtKB [51] (Table 1). According to homology relationships, we built four phylogenies corresponding to: i) all catalytic subunits; ii) class IA regulatory subunits; iii) class IB regulatory subunits; and iv) the class III regulatory protein. Metazoan homologs were retrieved from Ensembl [52] and other eukaryotic homologs were retrieved from a local database of complete proteomes. Similarity searches were performed on the two databases using BLASTP [53] with default settings and a cut-off set to *E*≤10^−30^. Because PI3K proteins have distant homologs, the retrieved homologs were used as the seed for new BLASTP runs with the same parameters (Additional file 1). To keep only one protein sequence per genomic locus, we grouped alternative transcripts together using the E-utilities [54] and the ACNUC sequence retrieval system [55]. Then, we manually selected the most conserved and/or the better aligned peptides.

In order to decrease noise and phylogenetic redundancy, we defined two taxonomic samplings. For the catalytic dataset, a subset of 44 representative species was selected for in-depth phylogenetic analysis. Among them, we kept only ten mammals over the 42 available in Ensembl. This choice was driven by three constraints: i) having a good representative diversity for the main eukaryotic groups; ii) limiting the number of fast-evolving sequences; and iii) including model organisms such as *S. cerevisiae* and *C. elegans*. For regulatory datasets, we only made a selection among mammals and kept all the other species.

For the multiple alignments we compared the results returned by PRANK [56] and MAFFT [57] using NorMD [58]. As its scores were consistently better, we chose to use the alignments computed by MAFFT. According to author recommendations, we set the maximum number of iterations at 100 and used the localpair options (equivalent to the linsi option). Alignments were then trimmed using BMGE [59]. Several sets of parameters were tested for this program in order to get a balance between the number of sites selected and the quality of the resulting multiple alignments (Additional file 2). Number of gaps per sequence after site selection are listed in Additional files 3 to 8.

The selection of evolutionary models used for the phylogenetic inference was carried out using ProtTest [60] and the Bayesian Information Criterion (BIC) [61]. In addition to the standard amino acids substitution models implemented in ProtTest we also performed the BIC test with UL3 [62] and CAT20 [63] models. The JTT+ *Γ*_4_ model [64] was proposed for the regulatory class IA and IB proteins, the subset for Opisthokonta homologs of class II and the complete catalytic subunits dataset. The UL3+ *Γ*_4_ model [62] was suggested for the regulatory class III, and for the reduced catalytic subunits datasets. Finally, the subset for Opisthokonta homologs of class I was inferred using the LG+ *Γ*_4_ model [65].

Maximum likelihood trees were built with PhyML [66]. Shape parameter of the Gamma distribution was estimated by PhyML with four categories for substitution rates. Branch statistical supports were estimated by the Shimodaira-Hasegawa-like test (SH) and non-parametric bootstrap (BS) with 1000 replicates.

A Bayesian approach was also used to infer the phylogeny of the eukaryotic dataset of catalytic subunits. For that purpose we used MrBayes [67]. Default parameters were used with the exception of the substitution model for amino acids, which was set to mixed. Seven million MCMC (Markov Chain Monte-Carlo) iterations were required to reach convergence. Burn-in values were set at 50 % of the iterations and we built a 50 % majority rule consensus tree after sampling one thousand trees from the posterior distribution. This sample was also used to establish the clades posterior probabilities (PP).

Domain composition analysis of all sequences was performed using HMMScan from the HMMER package [68, 69]. We searched for domains in both PfamA and PfamB databases and used all default parameters. For class IA regulatory proteins we also used Batch CD-Search [70] to confirm the presence of the p110 binding domain in non-Euteleostomi sequences.

## Results

### Phylogeny of PI3K

We applied a two-step strategy to decipher the evolutionary history of PI3Ks catalytic and regulatory subunits. First we investigated the taxonomic distribution of PI3Ks in all eukaryotes and constructed the corresponding phylogenies in order to identify the major evolutionary events that have affected these proteins during the diversification of eukaryotes. Then we performed a detailed analysis in Metazoa, Choanoflagellida and Ichthyosporea in order to investigate the pattern of duplications that led to the great expansion of this protein family in these lineages, including human.

#### Catalytic subunits

**Fig. 1:**
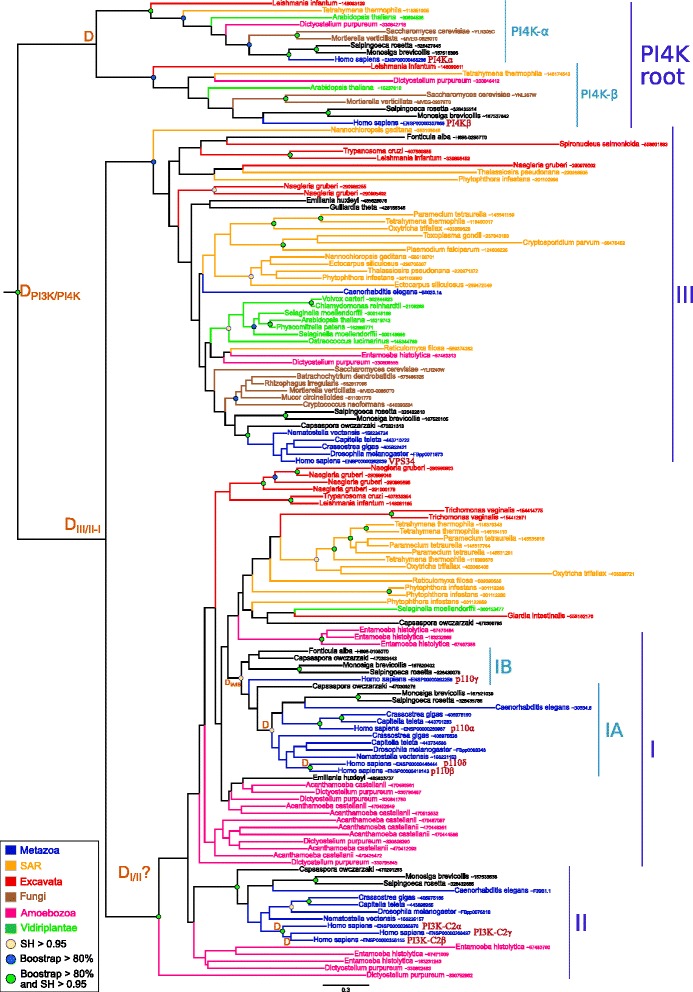
Maximum likelihood phylogenetic tree of catalytic PI3Ks subunits. The tree was inferred with the UL3+ *Γ*_4_ model (398 sites, 139 sequences). Sequences are colored according to their taxonomic classification. Green circles correspond to nodes with SH >0.95 and BS >80 *%*. Blue and yellow circles correspond to nodes having either SH >0.95 or BS >80 *%*. Duplication events are indicated by an orange “D”. The scale bar represents the average number of substitutions per site

For catalytic subunits, the multi seed similarity search performed allowed us to identify 1055 PI3K homologs. After a representative species selection (see Materials and methods), we reconstructed the maximum likelihood and Bayesian phylogenies of the 139 corresponding sequences. The resulting trees were congruent and in agreement with the phylogeny inferred with the complete set of 1055 sequences (Fig. 1 and Additional files 9 and 10). These trees showed two well supported clusters corresponding to class III and to classes I and II homologs, respectively (BS of 97 and 86 %, SH of 0.94 and 0.97, both PP of 1.0). Class III homologs are found in all major eukaryotic groups: SAR, Excavata, Archaeplastida, Amoebozoa and Opisthokonta (*i.e.*, Fungi, Metazoa and unicellular relatives). We also found sequences from Haptophyta (*Emiliana huxleyi)*, Cryptophyta (*Guillardia theta*) and Apusozoa (*Thecamonas trahens*). It is worth noting that no PI3K catalytic subunit was detected in red algae, while complete proteomes of three species were present in our database. The second cluster gathered sequences of classes I and II from all eukaryotic lineages with the exception of Fungi and most Archaeplastida (Fig. 1 and Additional files 9 and 10). Specific similarity searches in Archaeplastida and Fungi in the Non-Redundant NCBI database (NR) confirmed these absences (data not shown). Regarding other eukaryotic lineages, only one copy was present in Bikonta lineages (SAR, Excavata and Haptophyta), while two copies (corresponding to class I and class II) were found in Unikonta (Amoebozoan and Opisthokonta).

These results suggested that two successive gene duplication events occurred during the diversification of eukaryotes. The first one is very ancient and took place in an ancestor of all present day eukaryotes. It led to the separation of class III and classes I-II catalytic subunits. The second duplication event led to the separation of class I and II catalytic subunit. The grouping of Bikonta homologs with Unikonta class I proteins could suggest that this duplication event occurred also before LECA, but would imply that all Bikonta lineages have independently lost the gene coding for the class II catalytic subunit. However, the grouping of Bikonta sequences with Unikonta class I sequences was not significantly supported (BS<80 *%*, SH<0.95 and PP<0.5). This allows another interpretation, in which the duplication event occurred in the ancestor of Unikonta and is thus more recent (Additional file 9). This scenario is more parsimonious regarding the number of losses. Depending on the scenario, LECA had three or two PI3K catalytic coding genes. In any case, three major independent loss events occurred during the diversification of eukaryotes: the class I/II in Archaeplastida and classes I and II in Fungi.

While gene duplication of PI3K catalytic subunits have been documented in animals (especially in humans), we highlighted similar situations in major eukaryotic groups as Excavata, Alveolata, Stramenopiles or Amoebozoa (Fig. 1). This indicated that the expansion of this gene family was specific to neither Metazoa nor multicellular organisms. In order to decipher in detail the evolutionary origin of PI3K catalytic subunits in Metazoa, we performed a phylogenetic analysis focused on this lineage using Choanoflagellida and Ichthyosporea as outgroups. No duplication events were found for the class III (data not shown). For the class II, we detected three paralogs (PI3K-C2 *α*, PI3K-C2 *β* and PI3K-C2 *γ*) in Vertebrata, as in Human, but only one copy in other metazoan species like Mollusca, Cnidaria or Arthropoda (Additional file 11). This indicated that duplication events, at the origin of the three human paralogs, occurred in Vertebrata (both SH>0.95). However, the three copies detected in *Petromyzon marinus* (*i.e.*, a Petromyzontidae) grouped with the PI3K-C2 *α* proteins, while the two copies of *Callorhincus milii* (*i.e.*, a Chondrichthye) grouped with the PI3K-C2 *α* and PI3K-C2 *β* proteins, respectively. In that case the surprising location of the Petromyzontidae sequences could be due to a high evolutionary rate for PI3K-C2 *γ* and PI3K-C2 *β* coding genes.

An alternative hypothesis could be that the duplication event at the origin of PI3K-C2 *β* and PI3K-C2 *γ* occurred in Gnathostomata, suggesting a loss and two independent duplications of a PI3K-C2 *α* coding gene in *P. marinus*. Theses two scenarios imply the loss of the PI3K-C2 *γ* in the chondrichthyen species. Testing these hypotheses would require more data from Chondrichthyes and Petromyzontidae.

For class I, different taxonomic distributions are observed for the subclasses IA and IB. In fact, we identified homologs of classes IA and IB in most Metazoa, Choanoflagellida, Ichthyosporea and one sequence of Nucleariidae (Fig. 1 and Additional file 12). This suggests that the common ancestor of MIC possessed both classes IA and IB catalytic subunits (SH=1 and BS>80 *%*) and that the duplication event occurred before MIC. However, we could not date more precisely this duplication event due to weak statistical supports in this part of the tree, and due to the small number of proteomes available for protists related to MIC (Nucleariidae and Apusozoa). Within Metazoa, the taxonomic distribution of class IB was narrower compared to class IA. While the former was found only in the sponge Amphimedon and in Chordata, the latter was found in some protostomian lineages (Annelida, Mollusca, Platyhelminthes and Arthropoda). This indicated that secondary losses of class IB occurred during the diversification of Metazoa.

A careful examination of the phylogeny of classes IA and IB revealed also several important duplication events (Additional file 12). Within class IA, p110 *δ* and p110 *β* were more closely related, while p110 *α* was more divergent. The tree suggested that the first duplication event occurred in the ancestor of Metazoa leading to the divergence of p110 *α*, while the separation of p110 *β* and p110 *δ* happened in the ancestor of Vertebrata (SH=1 and BS=100 *%*). As a consequence, the absence of p110 *α* in Arthropoda, Cnidaria and Placozoa (*Trichoplax adhaerens*) and the absence of the ancestral p110 *β*/p110 *δ* protein in Platyhelminthes should be interpreted as secondary losses. For class IB, the presence of two p110 *γ* in Actinopterygii and Chondrichthyes indicates that a duplication event occurred in the ancestor of the Gnathostomata but one of the two resulting paralogs was subsequently lost in Sarcopterygii explaining why only one p110 *γ* sequence is found in these lineages.

#### Regulatory subunits

**Fig. 2:**
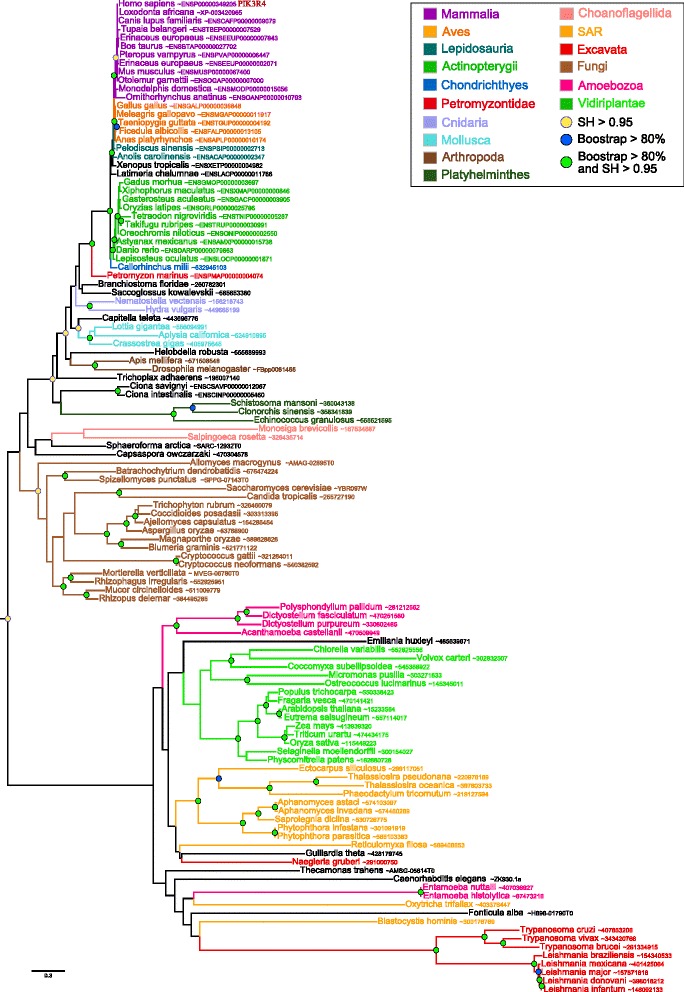
Maximum likelihood phylogenetic tree of class III regulatory PI3Ks subunits. The tree was inferred with the UL3+ *Γ*_4_ model (839 sites, 117 sequences). Sequences are colored according to their taxonomic classification. Branch statistical supports and duplication events are shown using the same symbols as in Fig. 1. The scale bar represents the average number of substitutions per site

For PI3K regulatory proteins, we found 117 homologs of the class III protein (VPS15), 126 homologs of the class IA and 67 homologs of the class IB protein. VPS15 homologs belonged to all the major eukaryotic groups including Fungi and Archaeplastida (Fig. 2). The taxonomic distribution and the maximum likelihood phylogeny of this protein were congruent with that of the catalytic class III subunit, indicating that both subunits were present in LECA and conserved along the diversification of present day eukaryotic lineages. The surprising grouping of sequences from *C. elegans* and *Fonticula alba* with Bikonta may be due to a long branch attraction artefact.

**Fig. 3:**
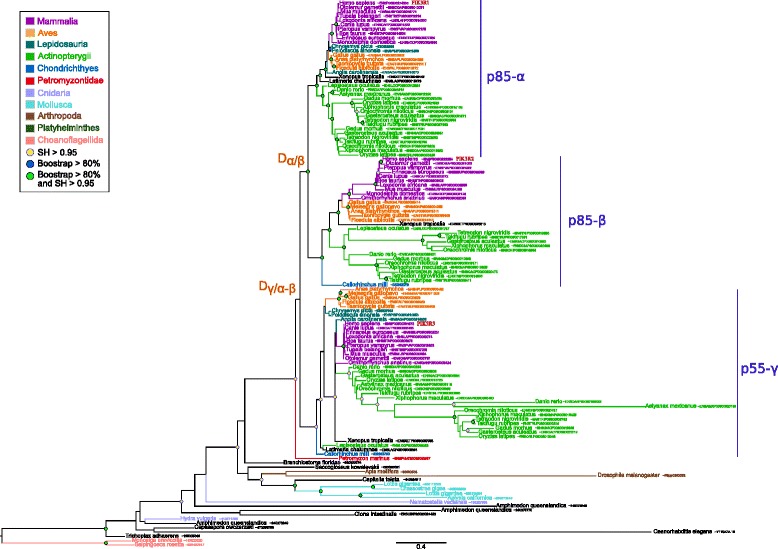
Maximum likelihood phylogenetic tree of class IA regulatory PI3Ks subunits. The tree was inferred with the JTT+ *Γ*_4_ model (539 sites, 126 sequences). Branch statistical supports and duplication events are shown using the same symbols as in Fig. 1. The scale bar represents the average number of substitutions per site

In contrast, regulatory subunits of class IA (p85 *α*, p85 *β* and p55 *γ*) showed a more restricted taxonomic distribution, being present only in MIC (Fig. 3). While the three proteins were found in Euteleostomi, p85 *β* and p85 *γ* were found also in Chondrichthyes. In contrast, a single protein was found in the other metaozan lineages, Ichtyosporea and Choanoflagellida. The phylogenetic analysis of these proteins strongly supported the grouping of p85 *α* and p85 *β* (BS=91 and SH=0.98). This suggested that the three human proteins derived from two Gnathostomata specific duplications followed by loss of the p85 *α* subunit in Chondrichthyes. However, as discussed before, because only one proteome was available for Chondrichthyes, we could not conclude with certainty about a loss in the whole taxonomic group. More surprisingly, we did not detect any p85 *β* ortholog in Lepidosauria.

**Fig. 4:**
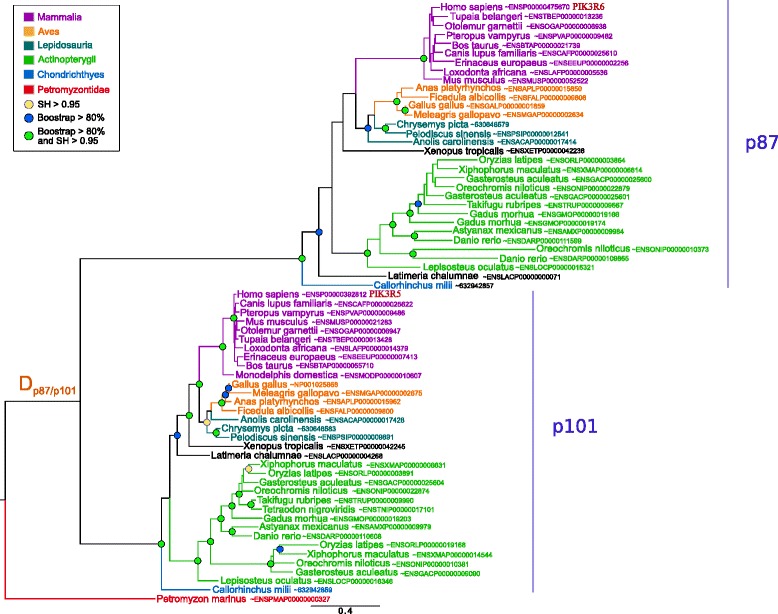
Maximum likelihood phylogenetic tree of class IB regulatory PI3Ks subunits. The tree was inferred with the JTT+ *Γ*_4_ model (599 sites, 67 sequences). Branch statistical supports and duplication events are shown using the same symbols as in Fig. 1. The scale bar represents the average number of substitutions per site

Finally, we determined that the two class IB regulatory subunits (p87 and p101) were homologous. Two paralogs were detected in Chondrichthyes, Sarcopterygii and Actinopterygii while only one sequence was present in Petromyzontidae. This strongly suggested that class IB emerged in the last common ancestor of Vertebrata and that a specific duplication underwent at the base of Gnathostomata (Fig. 4).

### Domain composition evolution

Our phylogenetic analyses revealed that PI3K proteins have a complex evolutionary history involving many lineage specific duplications and, to a lesser extent, losses. To get insights on the putative function of the PI3K proteins in non-model eukaryotic species, we performed a survey of their domain composition.

#### Catalytic subunits

**Fig. 5:**
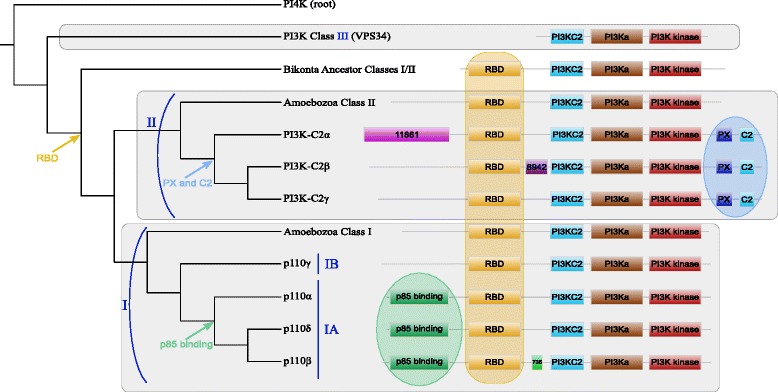
Domain composition of catalytic proteins. The schematic phylogenetic tree on the left corresponds to the complete tree of Fig. 1. Light colored circles indicate domains specific to some class I and II subgroups. Box sizes are not related to the real length of the domains

First, our results confirmed that all eukaryotic catalytic subunits shared three common domains in the same order: PI3KC2 (accession number PF00792), PI3KA (PF00613) and PI3K kinase (PF00454) (Fig. 5). Homologs of class III catalytic subunit did not harbour additional domains, while class I and II proteins possessed, in addition, the Ras Binding Domain (RBD, PF00794). This domain appeared after the class III catalytic diverged from the ancestral protein of classes I and II (*i.e.*, before LECA). It is essential for the activation of PI3K catalytic proteins by the Ras protein [71, 72]. This suggests that a functional change occurred after the duplication at the origin of classes III and I/II.

Amoebozoan class I and II proteins as well as Bikonta ancestor of class I/II protein shared exactly the same domain composition (*i.e.*, the four previously mentioned domains), precluding any conclusion regarding functional changes. In contrast the Opisthokonta subunits of classes I and II differed in their domain composition. First, we confirmed the specific presence of PX (PF00787) and C2 (PF00168) domains in class II catalytic proteins[73]. Furthermore, we detected two additional domains specific to class II PI3K-C2 *α* and PI3K-C2 *β* proteins: PB011861 was found at the N-terminal part of the PI3K-C2 *α* homologs, whereas PB008942 was located in-between the RBD and PI3KC2 domains of PI3K-C2 *β* homologs.

For class I, we confirmed the presence of the p85 binding domain (PF02192) in all IA homologs and its absence in IB homologs. Interestingly, the acquisition of the p85 binding domain by class IA proteins occurred in the last common ancestor of Metazoa, Ichthyosporea and Choanoflagellida, *i.e.*, while class IB diverged from class IA (see before). This coincided exactly with the origin of class IA regulatory proteins. This result was consistent with the fact that catalytic and regulatory subunits class IA form heterodimers through their p85 and p110 (or iSH2, PB011403) binding domains, respectively[74–78]. Among class IA catalytic proteins, p110 *β* has a specific PfamB domain (PB000735) located in-between RBD and PI3KC2 while p110 *α* and p110 *δ* share exactly the same domain composition (Fig. 5).

#### Regulatory subunits

**Fig. 6:**
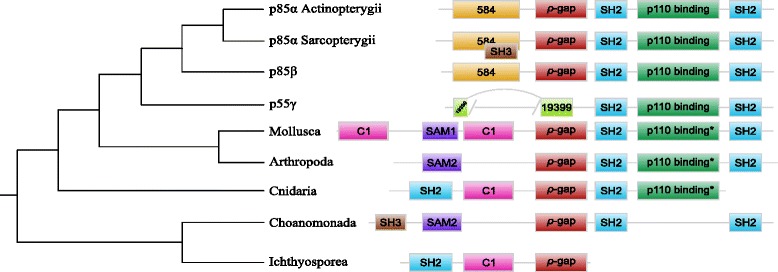
Domain composition of class IA regulatory proteins. The schematic phylogenetic tree on the left corresponds to the complete tree of Fig. 2. The presence of the p110 binding domains in non Osteichthyes sequences was assessed using Batch CD-search and are indicated with asterisks (*). Box sizes are not relative to the real length of the domains

All class IA regulatory subunits harboured the same C-terminal domain composition, *i.e.*, a *ρ*-gap domain (PF00620) followed by two SH2 (PF00017) domains intercut by a PB011403 (p110-binding) domain (Fig. 6). Three exceptions were the p55 *γ* homologs that lacked the *ρ*-gap domain but had a PB019399 domain, and the short Ichthyosporea and the Choanoflagellida proteins that lacked the p110-binding domain. This result is puzzling given that we detected class IA catalytic subunits in Ichthyosporea and Choanoflagellida (ancestor of p110 *α*- *β*- *δ*). This suggested that in these species, the p110 binding domain is not required for the interaction between the regulatory and catalytic subunits. Because Ichthyosporea were represented by a single species in our databases, we could wonder if the very short protein detected is real or is artifactual because of sequencing errors. In addition to the three conserved domains (*ρ*-gap, SH2 and PB011403), additional domains are present in the N-terminal of some sequences (Fig. 6). For instance, the p85 *α* and p85 *β* proteins contained a PB000584 domain, while the copy present in Mollusca, Cnidaria and Ichtyosporea have a C1 (PF00130) domain at this location. We detected SAM_1 (PF00536) or SAM_2 (PF07647) domains in Mollusca, Arthropoda and Choanoflagellida, whereas Cnidaria and Ichtyospoera harboured an additional SH2 domain. Finally, we detected SH3 (PF00018) in Choanoflagellida and in p85 *α* Actinopterygii homologs. Concerning class IB regulatory proteins, no domain was previously described. Our analysis detected only one PfamB domain named PI3K_1B_p101 (PF10486) in all dataset proteins (data not shown).

**Fig. 7:**
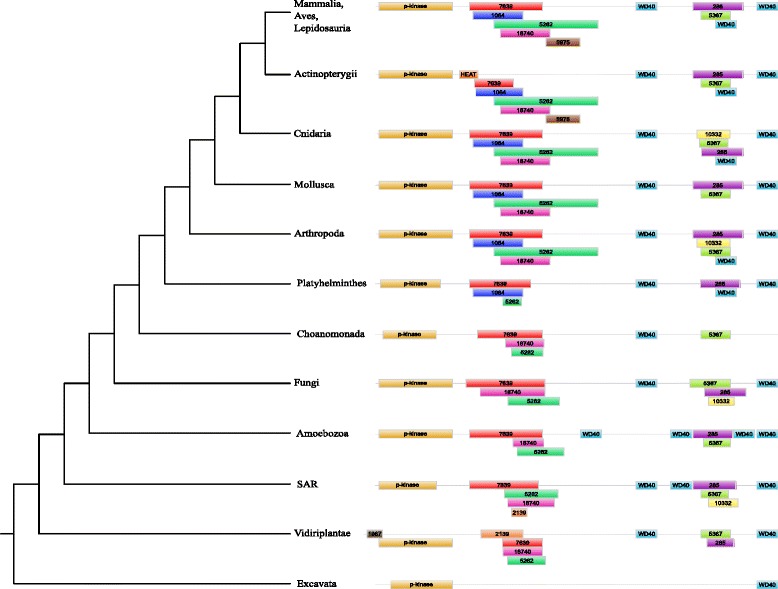
Domain composition of class III regulatory proteins. The schematic phylogenetic tree on the left corresponds to the complete tree of Fig. 4. Box sizes are not related to the real length of the domains

Finally, class III regulatory proteins showed a diverse domain composition (Fig. 7). The main information was that all proteins possessed a well-conserved Pkinase domain (PF00069) located at the N-terminal part of the proteins and two or more WD40 domains (PF00400) at their C-terminal part. Finally, a PB000285 domain, located between WD40 domains, was present in most of eukaryotic proteins except in Choanoflagellida and Excavata.

## Discussion

**Fig. 8:**
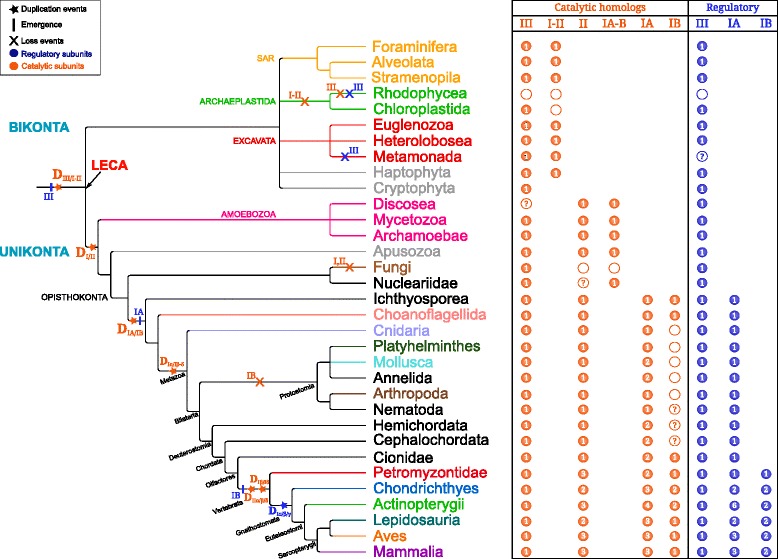
Schematic representation of PI3K evolutionary history. The eukaryotic tree was built according to Adl *et al.* [35], Delsuc *et al.* [87] papers and Lecointre and Le Guyader book [88]. Information about catalytic and regulatory subunits are displayed in orange and dark blue, respectively. Taxonomic group colors are the same as in Fig. 1. Number of protein copies are indicated in the colored circles and the absence of homologs resulting from secondary losses are represented by a dotted circle. The question marks correspond to supposed lack of homologs for taxonomic groups where only one proteome is available in our databases. Duplication events are indicated by a star, losses by a cross and the emergence of regulatory subunit by a vertical line

PI3K proteins are key players of cell signalling pathways. These proteins form a very ancient protein family in eukaryotes that can be traced back to LECA. The evolutionary history of this protein family was complex and involved a lot of gene duplications and losses (Fig. 8). In addition, substantial functional changes likely occurred through gains or losses of functional domains.

Our analyses showed that two paralogous catalytic PI3K were present in LECA (class III and I/II). This indicates that the corresponding duplication is ancient and occurred during the eukaryogenesis. The regulatory subunit class III was also present in LECA meaning that, at this time, the two class III proteins were present and likely interacted together. In human and yeast, the main biological function of class III proteins is to induce autophagy by regulating autophagosome formation [29–31]. This suggested that these processes could have been already established in LECA, which could be further investigated by the phylogenetic study of the other proteins involved in this crucial function.

In agreement with the previous studies [49, 50], we found that two major duplication events affected the evolutionary history of catalytic subunits. As Brown and Auger, we inferred that the first duplication leading to the separation of class III and classes I/II proteins occurred before LECA. In the case of the Kawashima *et al.* study, the data set was only made of sequences that came from five Opisthokonta species (*Homo sapiens*, *D. melanogaster*, *C. elegans*, *Ciona intestinalis* and *S. pombe*), therefore, their taxonomic sample was too restricted to conclude precisely the timing of this duplication.

We detected neither catalytic nor regulatory PI3Ks proteins in red algae, suggesting three independent gene losses in this lineage. Among the three complete proteomes present in our database (*Chondrus crispus*, *Cyanidioschyzon merolae* and *Galdieria sulphuraria*) the first harbour an unusual structure [79], and the others are very small for eukaryotic genomes [80, 81]. Further analyses are needed to confirm and explain these absences.

The taxonomic distribution and the phylogeny of class I/II catalytic subunits suggested that these two classes originated in the Unikonta lineage. This implied the presence of an ancestral class I/II protein in Bikonta lineages. In our study, as in Brown and Auger, these sequences grouped with the class I homologs. But unlike them, we found that this branch of the tree was not significantly supported (BS=7 *%* and SH=0.87). All non-Opisthokonta proteins from classes I and II have the same domain composition which do not help to infer different biochemical or molecular functions for these paralogs. Moreover, the ancestral class I/II protein present in LECA might have the same biological function as the protein present in all present day Bikonta organisms. Unfortunately, due to very few little functional studies available for Bikonta, we cannot make reasonable assumptions on the role of this protein in LECA. It would be interesting to study this protein in model organisms such as *Leishmania* and *Paramecium* species to infer its function in LECA.

In agreement with previous studies, we confirmed that *S. cerevisiae* possess only class III PI3K proteins [7, 50]. We extended this observation to all Fungi and showed that this resulted from independent losses of class I and II catalytic proteins in this lineage. Similarly, no Archaeplastida class I and II sequences were detected except in *Selaginella moellendorffii* in which a class I catalytic subunit was found. *S. moellendorffii* was not among the three Archaeplastida species of Brown and Auger dataset, so they did not find homologs in Archaeplastida in their study. Three hypotheses can explain the presence of a class I protein in *S. moellendorffii*: i) multiple and independent gene loss events occurred during the diversification of Archaeplastida except for this plant lineage; ii) an ancient gene loss event occurred in the common ancestor of Archaeplastida followed by a reacquisition by horizontal gene transfer in *S. moellendorffii*; iii) the sequence detected in this species was a contamination. In order to test the third hypothesis we performed a BLAST search to identify the homologs of the six protein genes surrounding the *S. moellendorffii* gene (data not shown). Best significant hits were all obtained with sequences from Viridiplantae, which invalidates this hypothesis of a contamination.

The evolutionary history of classes I and II was punctuated by gene duplications and losses. The two previous published phylogenies disagreed regarding the pattern of duplications. Brown and Auger found that p110 *α* and p110 *γ* (class IA and IB, respectively) grouped together, while Kawashima *et al.* found the three class IA proteins (p110 *α*, p110 *β* and p110 *δ*) in the same cluster. Our analyses agreed with the result of Kawashima *et al.*, but provided a more precise picture because we used 117 Opisthokonta complete proteomes, while they analyzed less than 30 Opisthokonta species. In fact, we found that a first duplication occurred before the last common ancestor of Metazoa, Ichthyosporea and Choanoflagellida and led to the separation of class IA and IB. Due to the low number of proteomes available for Nucleariidae and Apusozoa (only one of each in our database) and weak statistical branch supports, we could not date more precisely this gene duplication event. This question should be further addressed to conclude if the common ancestor of Opisthokonta possessed one or two copies of class I catalytic subunits. An Opisthokonta specific duplication would imply two independent gene losses in Fungi and one in Nucleariidae and Apusozoa, whereas a MIC specific duplication would only imply a gene loss in the fungal lineage and a misplacement of the Nucleariidae sequence. Then, two successive duplications occurred in class IA. The first one took place in the ancestor of Metazoa while the duplication leading to p110 *β* and p110 *δ* occurred in Vertebrata. The branch support of the subclasses duplication was not significant in the eukaryotic catalytic tree (SH=0.58, BS=38 *%* and PP=0.68), but both BS and SH values supported this node in the catalytic tree built with Ichthyosporea, Choanoflagellida and Metazoa homologs (Additional file 12). Moreover, the domain composition of those proteins – and especially the apparition of the p85-binding domain in class IA – supports the conjecture of a first duplication leading to the separation of the two subclasses before secondary duplications in subclass IA.

For class IA regulatory proteins, our results differed from the only comparable phylogeny available [49]. In fact, we found p85 *α* grouped with p85 *β* whereas Kawashima *et al.* found p85 *α* next to p55 *γ*. Nevertheless, corresponding branches were supported neither in their study nor in our phylogeny. More precisely, the first duplication event was well supported in Kawashima *et al.* (BS=100 *%*) but not in our study (SH=0.43 and BS=26 *%*). On the contrary, the second duplication event was supported by both values in our trees (SH=0.99 and BS=91 *%*), while the BS value was only equal to 82 % in the Kawashima *et al.* study. Interestingly, we detected class IA regulatory homologous proteins in Metazoa, Choanoflagellida and Ichtyosporea that exactly corresponds to the emergence of class IA catalytic subunits and the appearance of the p85-binding domain. In contrast, regulatory protein duplications occurred before the duplication of catalytic subunits (in Gnathostomata and Metazoa, respectively). So, in non-Gnathostomata organisms (*i.e.*, Mollusca, Annelida), there are two class IA catalytic subunits for only one class IA regulatory proteins. So we can hypothesize that the regulatory subunit of these organisms can regulate both p110 *α* and the ancestor of p110 *β*/p110 *δ* proteins or that the regulation is done by another protein not yet characterised.

For the catalytic class II, the two previous phylogenies found PI3K-C2 *α* grouped with the PI3K-C2 *β* while, in our trees, PI3K-C2 *β* is grouped with the PI3K-C2 *γ*. We found that these three proteins resulted from two successive duplications that occurred in the Vertebrata or Gnathostomata lineage. The discrepancy can be the consequence of a restricted taxonomic sampling and of less efficient methods (*i.e.*, neighbour-joining *vs.* maximum likelihood and Bayesian approaches). In terms of domain composition, proteins of classes I and II shared four specific domains. We confirmed the presence of both PX and C2 terminal domains [73, 82, 83] in all Opisthokonta class II proteins. We discovered that PI3K-C2 *α* and PI3K-C2 *β* shared a specific domain located in the first half of the sequence. This new information about these poorly understood catalytic subunits suggests that they had specific molecular or biochemical functions.

Furthermore, we provided a detailed phylogenetic analysis of class III protein (VPS15). Where Kawashima *et al.* used only two Fungi, one *Drosophila* and one *Ciona* species, we detected 117 homologous sequences belonging to all eukaryotic groups. Note that this ubiquity among eukaryotes was previously partially shown in [32]. Interestingly, no duplication event in any organism occurred during eukaryotic evolution for this class. Our results suggest that both catalytic and regulatory class III subunits were already present in LECA and conserved in one copy in Opisthokonta and other present-day eukaryotes (excepted *Naegleria gruberi* and some SAR which possessed two or more catalytic class III subunits). This contrasted with classes I and II PI3Ks.

We provided the first phylogenetic analysis of class IB regulatory proteins. We found that p87 and p101 proteins appeared very recently (in Vertebrata) and result from a specific Gnathostomata duplication. But the catalytic class IB protein emerged in the last common ancestor of Opisthokonta. This raises the question of the regulation of IB catalytic protein in other animals, Choanoflagellida and Ichthyosporea organisms.

Finally, in terms of biological functions, a lot of studies demonstrated the implication of class I proteins in chemotaxis in *Dyctiostelium* [39–41]. Interestingly, in human, class IB is involved in the chemotaxis of different cell types like macrophages [84] or smooth muscle and CD4^+^ T cells [85]. On the contrary, human class IA proteins are implicated in mitosis [86] and cell growth/proliferation through the AKT/mTOR signalling pathway regulation [7]. Accordingly, it is tempting to hypothesize that the ancestral function of class I was chemotaxy. Given that the duplication leading to classes IA and IB occurred in the Opisthokonta lineage, we can wonder if there is a link between the duplication and the emergence of multicellularity in this taxon.

## Conclusion

PI3Ks form a complex and very ancient protein family. This study allowed us to establish a much more accurate landscape of its evolutionary history thanks to the use of a broad set of completely sequenced eukaryotes. On the other hand, some parts of the trees we built for the different PI3K subunits are still poorly resolved. Especially we were unable to date precisely some duplication events (*e.g.*, duplication of the the three catalytic subunits of class II). This is mainly due to the lack of data for organisms such as Exacavates, SAR, Petromyzontidae and Chondrichthyes. Using the grounds provided by the approaches developed for this research, it will be possible to perform a broader study on the different proteins involved in the whole AKT/mTOR signaling pathway.

## Availability of supporting data

The different data sets supporting the results of this article (multiple sequence alignments) are available at http://pbil.univ-lyon1.fr/datasets/Philippon2015/.

## Additional file

Additional file 1
**Organisms used as seeds for the second BLAST search.** For detecting distant homologs we used sequences from 25 organisms as seeds for a second BLAST search. We choose organisms from different taxonomic groups in order to reach all eukaryotic homologs.

Additional file 2
**Datasets characteristics and program parameters used.** For each dataset, some information (number of human paralogs, number of homologs found, number of selected sites, etc.), as well as the parameters used for BMGE program and the substitution model selected are given. Also, the names of the supporting data files containing the corresponding trimmed multiple alignments are given.

Additional file 3
**Number of gaps per sequence after site selection for the reduced catalytic dataset.** Sequences are sorted by increased percentage of gaps.

Additional file 4
**Number of gaps per sequence after site selection for the regulatory subunit class III dataset.** Sequences are sorted by increased percentage of gaps.

Additional file 5
**Number of gaps per sequence after site selection for the regulatory subunit class IA dataset.** Sequences are sorted by increased percentage of gaps.

Additional file 6
**Number of gaps per sequence after site selection for the the regulatory subunit class IB dataset.** Sequences are sorted by increased percentage of gaps.

Additional file 7
**Number of gaps per sequence after site selection for the MIC class II catalytic subunit dataset.** Sequences are sorted by increased percentage of gaps.

Additional file 8
**Number of gaps per sequence after site selection for the MIC class I catalytic subunit dataset.** Sequences are sorted by increased percentage of gaps.

Additional file 9
**Complete phylogenetic tree of catalytic subunits.** The tree was inferred with the JTT+ *Γ*_4_ model (468 sites, 1055 sequences). Sequences are colored according to their taxonomic classification. SH support is indicated over the branches. Duplication events are shown by an orange“D”. The scale bar represents the average number of substitutions per site.

Additional file 10
**Bayesian phylogenetic tree of selected catalytic subunits.** The tree was inferred using the MrBayes program and the same alignment as the one used to build the corresponding maximum likelihood tree (Fig. 1). Sequences are colored according to their taxonomic classification. Yellow and red circles correspond to PP >0.90 and PP >0.95, respectively. Duplication events are indicated by an orange “D”. The scale bar represents the average number of substitutions per site.

Additional file 11
**Phylogenetic tree of Metazoa, Ichthyosporea and Choanoflagellida homologs of class II catalytic proteins.** The tree was inferred with the JTT+ *Γ*_4_ model (1113 sites, 108 sequences). Sequences are colored according to their taxonomic classification. Branch statistical supports and duplication events are shown using the same symbols as in Fig. 1. As described in the Material and methods section, we selected all non-mammal species from Ensembl, and kept ten representative mammal organisms and all Ichtyosporea, Choanoflagellida and Metazoa species from our local database.

Additional file 12
**Phylogenetic tree of Metazoa, Ichtyosporea and Choanoflagellida homologs of PI3K class I catalytic proteins.** The tree was inferred with the LG+ *Γ*_4_ model (828 sites, 185 sequences). Sequences are colored according to their taxonomic classification. Branch statistical supports and duplication events are shown using the same symbols as in Fig. 1.

## Abbreviations

BIC: Bayesian Information Criterion
BS: Bootstrap support
CAT: Categories model
GPCR: G Protein-Coupled Receptors
IGF1: Insulin-like Growth Factor 1
JTT: Jones, Taylor and Thornton model
LECA: Last Eukaryotic Common Ancestor
LG: Le and Gascuel model
LPA: Lysophosphatidic Acid
MIC: Metazoa, Ichthyosporea and Choanoflagellida
NR: Non-Redundant (database)
PI: phosphoinositides
PI3K: Phosphatidylinositol-3-kinases
PP: posterior probability
PTEN: Phosphatase and Tensin homolog
RBD: Ras Binding Domain
RTK: Receptor Tyrosine Kinase
SAR: Stramenopiles, Alveolata and Rhizaria
SH: Shimodaira-Hasegawa like support
UL3: Three-matrix unsupervised model
VPS15: Vacuolar Protein Sorting 15 (also named PIK3R4)
VPS34: Vacuolar Protein Sorting 34 (also named PIK3C3)
