# Supplementary material for: Evolutionary history of phosphatidylinositol- 3-kinases: ancestral origin in eukaryotes and complex duplication patterns
Source: BMC Evol Biol. 2015 Oct 19;15:226. doi: 10.1186/s12862-015-0498-7 (PMC4617754; doi:10.1186/s12862-015-0498-7)
Supplement: Additional file 1 — Organisms used as seeds for the second BLAST search. For detecting distant homologs we used sequences from 25 organisms as seeds for a second BLAST search. We choose organisms from different taxonomic groups in order to reach all eukaryotic homologs. [file 12862_2015_498_MOESM1_ESM.pdf]

Species as seeds for second Blast	
Catalytic dataset	Regulatory datasets
Arabidopsis thaliana	Apis mellifera
Cryptococcus neoformans	Capitella teleta
Dictyostelium purpureum	Lottia gigantea
Ectocarpus siliculosus	Nematostella vectensis
Emiliania huxleyi	Trichoplax adhaerens
Encephalitozoon cuniculi	
Entamoeba histolytica	
Giardia intestinalis	
Guillardia theta	
Monosiga brevicollis	
Naegleria gruberi	
Ostreococcus lucimarinus	
Paramecium tetraurelia	
Perkinsus marinus	
Phytophthora infestans	
Plasmodium falciparum	
Rhizophagus irregularis	
Thalassiosira pseudonana	
Trypanosoma brucei	
Trypanosoma cruzi	
