# Supplementary material for: Evolutionary history of phosphatidylinositol- 3-kinases: ancestral origin in eukaryotes and complex duplication patterns
Source: BMC Evol Biol. 2015 Oct 19;15:226. doi: 10.1186/s12862-015-0498-7 (PMC4617754; doi:10.1186/s12862-015-0498-7)
Supplement: Additional file 2 — Datasets characteristics and program parameters used. For each dataset, some information (number of human paralogs, number of homologs found, number of selected sites, etc.), as well as the parameters used for BMGE program and the substitution model selected are given. Also, the names of the supporting data files containing the corresponding trimmed multiple alignments are given. [file 12862_2015_498_MOESM2_ESM.pdf]

Dataset	Number of human paralogs	Figure number	Number of homologs	BMGE parametrer			Number of selected sites	Model selected by BIC	Alignment file name
				BLOSUM matrix	Maximum gap rate allowed per character (%)	Minimum length of selected region			
Eukaryotic selected homologs of catalytic subunits	8	1	139	30	50	2	398	UL3+G	Eukaryotic_catalytic_subunits_selection.fasta
Regulatory subunit class III	1	2	117	30	40	3	839	UL3+G	Regulatory_class_III.fasta
Regulatory class IA subunits	3	3	126	30	40	4	539	JTT+G	Regulatory_class_IA.fasta
Regulatory class IB subunits	2	4	67	30	50	4	599	JTT+G	Regulatory_class_IB.fasta
Catalytic homologs total	8	S1	1055	30	70	2	468	JTT+G	Eukaryotic_catalytic_subunits.fasta
MIC class II catalytic subunits	3	S3	108	30	40	4	1113	JTT+G	MIC_class_II_catalytic.fasta
MIC class I catalytic subunits	4	S4	185	30	40	4	828	LG+G	MIC_class_I_catalytic.fasta
