# Supplementary material for: Evolutionary history of phosphatidylinositol- 3-kinases: ancestral origin in eukaryotes and complex duplication patterns
Source: BMC Evol Biol. 2015 Oct 19;15:226. doi: 10.1186/s12862-015-0498-7 (PMC4617754; doi:10.1186/s12862-015-0498-7)
Supplement: Additional file 3 — Number of gaps per sequence after site selection for the reduced catalytic dataset. Sequences are sorted by increased percentage of gaps. [file 12862_2015_498_MOESM3_ESM.pdf]

Organism name and sequence ID	Number of gaps (percentage)
Homo sapiens ENSP00000418143	0 (0.0)
Homo sapiens ENSP00000392258	0 (0.0)
Phytophthora infestans 301122859	0 (0.0)
Trichomonas vaginalis 154414775	0 (0.0)
Dictyostelium purpureum 330806390	0 (0.0)
Dictyostelium purpureum 330795845	0 (0.0)
Acanthamoeba castellanii 470452549	0 (0.0)
Capsaspora owczarzaki 470309276	0 (0.0)
Drosophila melanogaster FBpp0083348	0 (0.0)
Paramecium tetraurelia 145531291	0 (0.0)
Tetrahymena thermophila 118379343	0 (0.0)
Homo sapiens ENSP00000263967	0 (0.0)
Entamoeba histolytica 67467388	0 (0.0)
Entamoeba histolytica 67471009	0 (0.0)
Oxytricha trifallax 403365406	0 (0.0)
Capsaspora owczarzaki 470303443	0 (0.0)
Nematostella vectensis 156221153	0 (0.0)
Entamoeba histolytica 67475484	0 (0.0)
Dictyostelium purpureum 330790467	0 (0.0)
Acanthamoeba castellanii 470490961	0 (0.0)
Tetrahymena thermophila 146164110	0 (0.0)
Salpingoeca rosetta 326430078	0 (0.0)
Paramecium tetraurelia 145517764	0 (0.0)
Dictyostelium purpureum 330841780	0 (0.0)
Paramecium tetraurelia 145535818	0 (0.0)
Phytophthora infestans 301112280	0 (0.0)
Phytophthora infestans 301112286	0 (0.0)
Capitella teleta 443734586	0 (0.0)
Capitella teleta 443701283	0 (0.0)
Fonticula alba H696-01050T0	0 (0.0)
Crassostrea gigas 405975190	0 (0.0)
Naegleria gruberi 290980695	0 (0.0)
Tetrahymena thermophila 118399676	0 (0.0)
Monosiga brevicollis 167521039	0 (0.0)
Acanthamoeba castellanii 470446361	0 (0.0)
Trichomonas vaginalis 154412871	0 (0.0)
Reticulomyxa filosa 569390586	1 (0.25)
Caenorhabditis elegans F39B1.1	1 (0.25)
Homo sapiens ENSP00000356155	1 (0.25)
Leishmania infantum 146081165	1 (0.25)
Homo sapiens ENSP00000265970	1 (0.25)
Nematostella vectensis 156225157	1 (0.25)
Dictyostelium purpureum 330802463	1 (0.25)
Trypanosoma cruzi 407833354	1 (0.25)
Entamoeba histolytica 183231243	1 (0.25)
Capsaspora owczarzaki 470291293	1 (0.25)
Drosophila melanogaster FBpp0075818	2 (0.5)
Caenorhabditis elegans B0334.8	2 (0.5)
Salpingoeca rosetta 326432665	2 (0.5)
Capsaspora owczarzaki 470306795	3 (0.75)
Oxytricha trifallax 403336721	4 (1.01)
Naegleria gruberi 290990903	7 (1.76)
Homo sapiens ENSP00000446444	8 (2.01)
Giardia intestinalis 559182176	10 (2.51)
Entamoeba histolytica 67483780	10 (2.51)
Monosiga brevicollis 167533638	11 (2.76)
Emiliania huxleyi 485633737	13 (3.27)
Acanthamoeba castellanii 470425472	13 (3.27)
Homo sapiens ENSP00000266497	14 (3.52)
Naegleria gruberi 290975002	15 (3.77)
Oxytricha trifallax 403359629	17 (4.27)
Crassostrea gigas 405975165	17 (4.27)
Acanthamoeba castellanii 470512532	19 (4.77)
Entamoeba histolytica 183232689	21 (5.28)
Acanthamoeba castellanii 470444588	21 (5.28)
Fonticula alba H696-02957T0	22 (5.53)
Batrachochytrium dendrobatidis 575486326	22 (5.53)
Dictyostelium purpureum 330806555	22 (5.53)
Phytophthora infestans 301103680	22 (5.53)
Toxoplasma gondii 237843183	22 (5.53)
Salpingoeca rosetta 326432810	23 (5.78)
Caenorhabditis elegans B0025.1a	23 (5.78)
Emiliania huxleyi 485628976	23 (5.78)
Tetrahymena thermophila 118400017	23 (5.78)
Mucor circinelloides 511001770	23 (5.78)
Acanthamoeba castellanii 470412093	23 (5.78)
Paramecium tetraurelia 145541159	23 (5.78)
Physcomitrella patens 162685771	24 (6.03)
Arabidopsis thaliana 15219743	24 (6.03)
Crassostrea gigas 405952421	24 (6.03)
Rhizophagus irregularis 552917095	24 (6.03)
Capitella teleta 443710722	24 (6.03)
Nematostella vectensis 156224724	24 (6.03)
Homo sapiens ENSP00000262039	24 (6.03)
Saccharomyces cerevisiae YLR240W	24 (6.03)
Capsaspora owczarzaki 470321313	24 (6.03)
Mortierella verticillata MVEG-00850T0	24 (6.03)
Drosophila melanogaster FBpp0071973	24 (6.03)
Selaginella moellendorffii 300148168	25 (6.28)
Leishmania infantum 339898452	25 (6.28)
Plasmodium falciparum 124506225	25 (6.28)
Cryptosporidium parvum 66475452	25 (6.28)
Entamoeba histolytica 67483313	26 (6.53)
Selaginella moellendorffii 300148666	26 (6.53)
Trypanosoma cruzi 407860885	26 (6.53)
Cryptococcus neoformans 540393534	26 (6.53)
Nannochloropsis gaditana 585106701	27 (6.78)
Naegleria gruberi 290989255	27 (6.78)
Spironucleus salmonicida 558601893	28 (7.04)
Dictyostelium purpureum 330792962	29 (7.29)
Acanthamoeba castellanii 470467087	30 (7.54)
Phytophthora infestans 301100996	31 (7.79)
Ectocarpus siliculosus 299472549	32 (8.04)
Crassostrea gigas 405975626	33 (8.29)
Ectocarpus siliculosus 298705307	38 (9.55)
Dictyostelium purpureum 330842718	44 (11.06)
Reticulomyxa filosa 569374282	47 (11.81)
Mortierella verticillata MVEG-08250T0	48 (12.06)
Homo sapiens ENSP00000458238	49 (12.31)
Guillardia theta 428168346	49 (12.31)
Arabidopsis thaliana 30694536	50 (12.56)
Leishmania infantum 146093129	51 (12.81)
Tetrahymena thermophila 118351905	51 (12.81)
Salpingoeca rosetta 326427845	52 (13.07)
Naegleria gruberi 290999046	57 (14.32)
Naegleria gruberi 291000178	57 (14.32)
Ostreococcus lucimarinus 145344789	62 (15.58)
Monosiga brevicollis 167516396	64 (16.08)
Capitella teleta 443696965	64 (16.08)
Naegleria gruberi 290995492	68 (17.09)
Thalassiosira pseudonana 220971372	68 (17.09)
Chlamydomonas reinhardtii 2109289	69 (17.34)
Volvox carteri 302844923	69 (17.34)
Dictyostelium purpureum 330846412	77 (19.35)
Saccharomyces cerevisiae YNL267W	80 (20.1)
Mortierella verticillata MVEG-09379T0	84 (21.11)
Salpingoeca rosetta 326435514	85 (21.36)
Tetrahymena thermophila 146174543	86 (21.61)
Arabidopsis thaliana 15237610	88 (22.11)
Leishmania infantum 146099611	88 (22.11)
Monosiga brevicollis 167537642	88 (22.11)
Selaginella moellendorffii 300153477	88 (22.11)
Homo sapiens ENSP00000357869	89 (22.36)
Salpingoeca rosetta 326435786	89 (22.36)
Saccharomyces cerevisiae YLR305C	91 (22.86)
Nannochloropsis gaditana 585108648	148 (37.19)
Monosiga brevicollis 167520105	179 (44.97)
Thalassiosira pseudonana 220968505	204 (51.26)
Monosiga brevicollis 167520402	215 (54.02)
