# Supplementary material for: Evolutionary history of phosphatidylinositol- 3-kinases: ancestral origin in eukaryotes and complex duplication patterns
Source: BMC Evol Biol. 2015 Oct 19;15:226. doi: 10.1186/s12862-015-0498-7 (PMC4617754; doi:10.1186/s12862-015-0498-7)
Supplement: Additional file 4 — Number of gaps per sequence after site selection for the regulatory subunit class III dataset. Sequences are sorted by increased percentage of gaps. [file 12862_2015_498_MOESM4_ESM.pdf]

| Organism name and sequence ID | Number of gaps (percentage) |
|---|---|
| Spizellomyces punctatus SPPG-07143T0 | 0 (0.0) |
| Danio rerio ENSDARP00000079663 | 0 (0.0) |
| Gasterosteus aculeatus ENSGACP00000003905 | 0 (0.0) |
| Takifugu rubripes ENSTRUP00000030991 | 0 (0.0) |
| Tetraodon nigroviridis ENSTNIP00000005287 | 0 (0.0) |
| Xiphophorus maculatus ENSXMAP00000000846 | 0 (0.0) |
| Latimeria chalumnae ENSLACP00000011766 | 0 (0.0) |
| Drosophila melanogaster FBpp0081466 | 0 (0.0) |
| Callorhinchus milii 632945103 | 0 (0.0) |
| Branchiostoma floridae 260782301 | 0 (0.0) |
| Capitella teleta 443696776 | 0 (0.0) |
| Apis mellifera 571508548 | 0 (0.0) |
| Thecamonas trahens AMSG-05614T0 | 0 (0.0) |
| Batrachochytrium dendrobatidis 575474224 | 0 (0.0) |
| Crassostrea gigas 405975645 | 0 (0.0) |
| Lottia gigantea 556094991 | 0 (0.0) |
| Saprolegnia diclina 530726775 | 0 (0.0) |
| Aphanomyces astaci 574103097 | 0 (0.0) |
| Canis lupus ENSCAFP00000009079 | 0 (0.0) |
| Ornithorhynchus anatinus ENSOANP00000010793 | 0 (0.0) |
| Bos taurus ENSBTAP00000027702 | 0 (0.0) |
| Pteropus vampyrus ENSPVAP00000006447 | 0 (0.0) |
| Homo sapiens ENSP00000349205 | 0 (0.0) |
| Otolemur garnettii ENSOGAP00000007000 | 0 (0.0) |
| Mus musculus ENSMUSP00000067400 | 0 (0.0) |
| Pelodiscus sinensis ENSPSIP00000002713 | 0 (0.0) |
| Anas platyrhynchos ENSAPLP00000010174 | 0 (0.0) |
| Ficedula albicollis ENSFALP00000013105 | 0 (0.0) |
| Taeniopygia guttata ENSTGUP00000004192 | 0 (0.0) |
| Xenopus tropicalis ENSXETP00000004982 | 0 (0.0) |
| Lepisosteus oculatus ENSLOCP00000001871 | 0 (0.0) |
| Oreochromis niloticus ENSONIP00000002550 | 1 (0.12) |
| Gadus morhua ENSGMOP00000003697 | 1 (0.12) |
| Ciona intestinalis ENSCINP00000005460 | 1 (0.12) |
| Nematostella vectensis 156218743 | 1 (0.12) |
| Aphanomyces invadans 574480269 | 1 (0.12) |
| Mucor circinelloides 511009779 | 2 (0.24) |
| Trichophyton rubrum 326460079 | 4 (0.48) |
| Aspergillus oryzae 83768900 | 4 (0.48) |
| Capsaspora owczarzaki 470304578 | 4 (0.48) |
| Coccidioides posadasii 303313395 | 4 (0.48) |
| Cryptococcus neoformans 540382592 | 5 (0.6) |
| Allomyces macrogynus AMAG-02895T0 | 6 (0.72) |
| Cryptococcus gattii 321264011 | 6 (0.72) |
| Helobdella robusta 555689993 | 6 (0.72) |
| Dictyostelium purpureum 330802485 | 7 (0.83) |
| Magnaporthe oryzae 389628626 | 7 (0.83) |
| Phaeodactylum tricornutum 219127594 | 7 (0.83) |
| Candida tropicalis 255727190 | 7 (0.83) |
| Phytophthora parasitica 568103383 | 9 (1.07) |
| Phytophthora infestans 301091919 | 9 (1.07) |
| Echinococcus granulosus 556521595 | 9 (1.07) |
| Blumeria graminis 521771122 | 9 (1.07) |
| Naegleria gruberi 291000750 | 9 (1.07) |
| Dictyostelium fasciculatum 470251580 | 10 (1.19) |
| Clonorchis sinensis 358341839 | 12 (1.43) |
| Fragaria vesca 470141421 | 13 (1.55) |
| Aplysia californica 524910995 | 14 (1.67) |
| Zea mays 413939320 | 14 (1.67) |

| Organism name and sequence ID | Number of gaps (percentage) |
|---|---|
| Physcomitrella patens 162680728 | 15 (1.79) |
| Selaginella moellendorffii 300154027 | 15 (1.79) |
| Eutrema salsugineum 557114017 | 15 (1.79) |
| Arabidopsis thaliana 15233564 | 15 (1.79) |
| Ostreococcus lucimarinus 145345011 | 16 (1.91) |
| Entamoeba histolytica 67473216 | 16 (1.91) |
| Entamoeba nuttalli 407036927 | 16 (1.91) |
| Monodelphis domestica ENSMODP00000015056 | 16 (1.91) |
| Acanthamoeba castellanii 470509949 | 17 (2.03) |
| Caenorhabditis elegans ZK930.1a | 19 (2.26) |
| Triticum urartu 474434175 | 19 (2.26) |
| Micromonas pusilla 303271833 | 25 (2.98) |
| Leishmania braziliensis 154340533 | 26 (3.1) |
| Mortierella verticillata MVEG-06780T0 | 29 (3.46) |
| Thalassiosira pseudonana 220976169 | 30 (3.58) |
| Fonticula alba H696-01790T0 | 33 (3.93) |
| Chlorella variabilis 552825556 | 33 (3.93) |
| Monosiga brevicollis 167534887 | 36 (4.29) |
| Populus trichocarpa 550336423 | 37 (4.41) |
| Schistosoma mansoni 360043138 | 47 (5.6) |
| Saccharomyces cerevisiae YBR097W | 50 (5.96) |
| Meleagris gallopavo ENSMGAP00000011917 | 51 (6.08) |
| Trypanosoma cruzi 407853206 | 54 (6.44) |
| Anolis carolinensis ENSACAP00000002347 | 59 (7.03) |
| Trypanosoma brucei 261334915 | 60 (7.15) |
| Tupaia belangeri ENSTBEP00000007529 | 64 (7.63) |
| Salpingoeca rosetta 326435714 | 69 (8.22) |
| Hydra vulgaris 449665199 | 72 (8.58) |
| Leishmania major 157871818 | 76 (9.06) |
| Leishmania infantum 146092133 | 76 (9.06) |
| Oxytricha trifallax 403376447 | 76 (9.06) |
| Leishmania donovani 398018212 | 76 (9.06) |
| Leishmania mexicana 401425064 | 76 (9.06) |
| Coccomyxa subellipsoidea 545368922 | 87 (10.37) |
| Loxodonta africana XP003420965 | 116 (13.83) |
| Ciona savignyi ENSCSAVP00000012067 | 124 (14.78) |
| Trichoplax adhaerens 196007140 | 127 (15.14) |
| Emiliania huxleyi 485639671 | 136 (16.21) |
| Astyanax mexicanus ENSAMXP00000015738 | 171 (20.38) |
| Saccoglossus kowalevskii 585653380 | 175 (20.86) |
| Ajellomyces capsulatus 154286454 | 175 (20.86) |
| Polysphondylium pallidum 281212562 | 179 (21.33) |
| Volvox carteri 302832507 | 183 (21.81) |
| Ectocarpus siliculosus 299117051 | 214 (25.51) |
| Rhizophagus irregularis 552925951 | 235 (28.01) |
| Erinaceus europaeus ENSEEUP00000007843 | 253 (30.15) |
| Gallus gallus ENSGALP00000036848 | 267 (31.82) |
| Rhizopus delemar 384495285 | 374 (44.58) |
| Erinaceus europaeus ENSEEUP00000002071 | 400 (47.68) |
| Blastocystis hominis 300176769 | 432 (51.49) |
| Reticulomyxa filosa 569406653 | 481 (57.33) |
| Petromyzon marinus ENSPMAP00000004074 | 534 (63.65) |
| Oryza sativa 115449223 | 588 (70.08) |
| Oryzias latipes ENSORLP00000025786 | 595 (70.92) |
| Guillardia theta 428179745 | 599 (71.39) |
| Thalassiosira oceanica 397603733 | 600 (71.51) |
| Sphaeroforma arctica SARC-12932T0 | 612 (72.94) |
| Trypanosoma vivax 343420766 | 672 (80.1) |
