# Supplementary material for: Evolutionary history of phosphatidylinositol- 3-kinases: ancestral origin in eukaryotes and complex duplication patterns
Source: BMC Evol Biol. 2015 Oct 19;15:226. doi: 10.1186/s12862-015-0498-7 (PMC4617754; doi:10.1186/s12862-015-0498-7)
Supplement: Additional file 5 — Number of gaps per sequence after site selection for the regulatory subunit class IA dataset. Sequences are sorted by increased percentage of gaps. [file 12862_2015_498_MOESM5_ESM.pdf]

Organism name and sequence ID	Number of gaps (percentage)
Callorhinchus milii 632962079	0 (0.0)
Xiphophorus maculatus ENSXMAP00000018652	0 (0.0)
Gasterosteus aculeatus ENSGACP00000020881	0 (0.0)
Takifugu rubripes ENSTRUP00000018795	0 (0.0)
Oreochromis niloticus ENSONIP00000013929	0 (0.0)
Takifugu rubripes ENSTRUP00000027603	0 (0.0)
Oryzias latipes ENSORLP00000021829	0 (0.0)
Danio rerio ENSDARP00000056212	0 (0.0)
Astyanax mexicanus ENSAMXP00000012290	0 (0.0)
Lepisosteus oculatus ENSLOCP00000012884	0 (0.0)
Ficedula albicollis ENSFALP00000009923	0 (0.0)
Anas platyrhynchos ENSAPLP00000006492	0 (0.0)
Meleagris gallopavo ENSMGAP00000011022	0 (0.0)
Gallus gallus ENSGALP00000023820	0 (0.0)
Lepisosteus oculatus ENSLOCP00000006953	0 (0.0)
Chrysemys picta 530627063	1 (0.19)
Callorhinchus milii 632963753	1 (0.19)
Oreochromis niloticus ENSONIP00000002191	1 (0.19)
Xiphophorus maculatus ENSXMAP00000015169	1 (0.19)
Tetraodon nigroviridis ENSTNIP00000022063	1 (0.19)
Gasterosteus aculeatus ENSGACP00000001371	1 (0.19)
Lepisosteus oculatus ENSLOCP00000001757	1 (0.19)
Otolemur garnettii ENSOGAP00000001020	1 (0.19)
Monodelphis domestica ENSMODP00000024566	2 (0.37)
Taeniopygia guttata ENSTGUP00000003114	2 (0.37)
Chrysemys picta 530622965	2 (0.37)
Ficedula albicollis ENSFALP00000010272	2 (0.37)
Gallus gallus ENSGALP00000023822	2 (0.37)
Pelodiscus sinensis ENSPSIP00000011890	2 (0.37)
Anolis carolinensis ENSACAP00000016070	2 (0.37)
Latimeria chalumnae ENSLACP00000015476	2 (0.37)
Homo sapiens ENSP00000274335	2 (0.37)
Gadus morhua ENSGMOP00000017031	2 (0.37)
Bos taurus ENSBTAP00000014594	2 (0.37)
Loxodonta africana ENSLAFP00000015303	2 (0.37)
Danio rerio ENSDARP00000056821	2 (0.37)
Oreochromis niloticus ENSONIP00000013171	2 (0.37)
Takifugu rubripes ENSTRUP00000026411	2 (0.37)
Pteropus vampyrus ENSPVAP00000007375	2 (0.37)
Gallus gallus ENSGALP00000005414	2 (0.37)
Ficedula albicollis ENSFALP00000014443	2 (0.37)
Canis lupus ENSCAFP00000011322	2 (0.37)
Mus musculus ENSMUSP00000056774	2 (0.37)
Tupaia belangeri ENSTBEP00000012016	3 (0.56)
Xenopus tropicalis ENSXETP00000046442	3 (0.56)
Oryzias latipes ENSORLP00000003326	3 (0.56)
Tetraodon nigroviridis ENSTNIP00000014006	3 (0.56)
Anas platyrhynchos ENSAPLP00000011511	3 (0.56)
Anas platyrhynchos ENSAPLP00000004356	4 (0.74)
Gadus morhua ENSGMOP00000003534	4 (0.74)
Gadus morhua ENSGMOP00000010892	5 (0.93)
Xiphophorus maculatus ENSXMAP00000013925	5 (0.93)
Ornithorhynchus anatinus ENSOANP00000002083	6 (1.11)
Monodelphis domestica ENSMODP00000002126	6 (1.11)
Mus musculus ENSMUSP00000034296	6 (1.11)
Homo sapiens ENSP00000222254	6 (1.11)
Loxodonta africana ENSLAFP00000000599	6 (1.11)
Canis lupus ENSCAFP00000022072	6 (1.11)
Taeniopygia guttata ENSTGUP00000008408	6 (1.11)
Saccoglossus kowalevskii 585680651	7 (1.3)
Pteropus vampyrus ENSPVAP00000001922	8 (1.48)
Bos taurus ENSBTAP00000003033	8 (1.48)
Branchiostoma floridae 260823974	9 (1.67)
Xenopus tropicalis ENSXETP00000028313	9 (1.67)
Apis mellifera 66500538	10 (1.86)
Gasterosteus aculeatus ENSGACP00000020473	11 (2.04)
Taeniopygia guttata ENSTGUP00000000409	14 (2.6)
Crassostrea gigas 405968860	34 (6.31)
Capitella teleta 443694211	34 (6.31)
Amphimedon queenslandica 340378846	36 (6.68)
Otolemur garnettii ENSOGAP00000013371	57 (10.58)
Salpingoeca rosetta 326427517	63 (11.69)
Meleagris gallopavo ENSMGAP00000004955	74 (13.73)
Oreochromis niloticus ENSONIP00000023417	101 (18.74)
Erinaceus europaeus ENSEEUP00000007255	115 (21.34)
Petromyzon marinus ENSPMAT00000003687	121 (22.45)
Oryzias latipes ENSORLP00000013359	121 (22.45)
Gasterosteus aculeatus ENSGACP00000006864	122 (22.63)
Oreochromis niloticus ENSONIP00000009899	122 (22.63)
Oryzias latipes ENSORLP00000016725	122 (22.63)
Ornithorhynchus anatinus ENSOANP00000009424	124 (23.01)
Xenopus tropicalis ENSXETP00000007065	124 (23.01)
Anolis carolinensis ENSACAP00000015633	124 (23.01)
Bos taurus ENSBTAP00000003878	124 (23.01)
Loxodonta africana ENSLAFP00000003074	124 (23.01)
Homo sapiens ENSP00000361075	124 (23.01)
Canis lupus ENSCAFP00000006438	124 (23.01)
Mus musculus ENSMUSP00000030464	124 (23.01)
Gadus morhua ENSGMOP00000000809	124 (23.01)
Xiphophorus maculatus ENSXMAP00000018429	124 (23.01)
Amphimedon queenslandica 340375770	125 (23.19)
Tupaia belangeri ENSTBEP00000007253	125 (23.19)
Pteropus vampyrus ENSPVAP00000012903	125 (23.19)
Gasterosteus aculeatus ENSGACP00000021319	125 (23.19)
Danio rerio ENSDARP00000040333	127 (23.56)
Gadus morhua ENSGMOP00000012642	127 (23.56)
Tetraodon nigroviridis ENSTNIP00000009742	133 (24.68)
Takifugu rubripes ENSTRUP00000030226	133 (24.68)
Pelodiscus sinensis ENSPSIP00000012953	137 (25.42)
Erinaceus europaeus ENSEEUP00000008939	143 (26.53)
Takifugu rubripes ENSTRUP00000000305	143 (26.53)
Otolemur garnettii ENSOGAP00000005767	146 (27.09)
Drosophila melanogaster FBpp0303632	151 (28.01)
Xiphophorus maculatus ENSXMAP00000005400	151 (28.01)
Tetraodon nigroviridis ENSTNIP00000013096	159 (29.5)
Takifugu rubripes ENSTRUP00000017331	159 (29.5)
Xiphophorus maculatus ENSXMAP00000002496	160 (29.68)
Oreochromis niloticus ENSONIP00000015985	160 (29.68)
Aplysia californica 524870249	161 (29.87)
Gasterosteus aculeatus ENSGACP00000010853	162 (30.06)
Latimeria chalumnae ENSLACP00000008684	165 (30.61)
Lottia gigantea 556112584	168 (31.17)
Caenorhabditis elegans Y110A7A.10	181 (33.58)
Erinaceus europaeus ENSEEUP00000002251	186 (34.51)
Danio rerio ENSDARP00000106632	258 (47.87)
Nematostella vectensis 156227304	301 (55.84)
Astyanax mexicanus ENSAMXP00000020188	308 (57.14)
Astyanax mexicanus ENSAMXP00000003118	368 (68.27)
Lottia gigantea 556112583	385 (71.43)
Ciona intestinalis ENSCINP00000024453	390 (72.36)
Tetraodon nigroviridis ENSTNIP00000022692	391 (72.54)
Capsaspora owczarzaki 470298756	396 (73.47)
Amphimedon queenslandica 340373849	397 (73.65)
Hydra vulgaris 449671265	399 (74.03)
Trichoplax adhaerens 196000346	401 (74.4)
Monosiga brevicollis 167520620	450 (83.49)
