# Supplementary material for: Evolutionary history of phosphatidylinositol- 3-kinases: ancestral origin in eukaryotes and complex duplication patterns
Source: BMC Evol Biol. 2015 Oct 19;15:226. doi: 10.1186/s12862-015-0498-7 (PMC4617754; doi:10.1186/s12862-015-0498-7)
Supplement: Additional file 6 — Number of gaps per sequence after site selection for the the regulatory subunit class IB dataset. Sequences are sorted by increased percentage of gaps. [file 12862_2015_498_MOESM6_ESM.pdf]

Organism name and sequence ID	Number of gaps (percentage)
Pteropus vampyrus ENSPVAP00000009486	0 (0.0)
Monodelphis domestica ENSMODP00000010607	0 (0.0)
Canis lupus ENSCAFP00000025622	0 (0.0)
Oreochromis niloticus ENSONIP00000022874	0 (0.0)
Takifugu rubripes ENSTRUP00000009990	0 (0.0)
Danio rerio ENSDARP00000110608	0 (0.0)
Astyanax mexicanus ENSAMXP00000009979	0 (0.0)
Gasterosteus aculeatus ENSGACP00000009090	0 (0.0)
Lepisosteus oculatus ENSLOCP00000016346	0 (0.0)
Loxodonta africana ENSLAFP00000014379	0 (0.0)
Mus musculus ENSMUSP00000021283	0 (0.0)
Chrysemys picta 530646583	0 (0.0)
Otolemur garnettii ENSOGAP00000006947	0 (0.0)
Homo sapiens ENSP00000392812	0 (0.0)
Ficedula albicollis ENSFALP00000009800	1 (0.17)
Gallus gallus NP001025868	1 (0.17)
Anas platyrhynchos ENSAPLP00000015962	1 (0.17)
Xiphophorus maculatus ENSXMAP00000006631	1 (0.17)
Gasterosteus aculeatus ENSGACP00000025604	1 (0.17)
Xiphophorus maculatus ENSXMAP00000014544	1 (0.17)
Bos taurus ENSBTAP00000055710	1 (0.17)
Oryzias latipes ENSORLP00000019168	1 (0.17)
Anolis carolinensis ENSACAP00000017428	2 (0.33)
Pelodiscus sinensis ENSPSIP00000009891	3 (0.5)
Oryzias latipes ENSORLP00000003891	4 (0.67)
Callorhinchus milii 632942859	4 (0.67)
Meleagris gallopavo ENSMGAP00000002675	5 (0.83)
Xenopus tropicalis ENSXETP00000042245	5 (0.83)
Gadus morhua ENSGMOP00000019203	5 (0.83)
Oreochromis niloticus ENSONIP00000010381	12 (2.0)
Petromyzon marinus ENSPMAP00000000327	16 (2.67)
Danio rerio ENSDARP00000111599	44 (7.35)
Otolemur garnettii ENSOGAP00000006938	44 (7.35)
Xenopus tropicalis ENSXETP00000042238	45 (7.51)
Anas platyrhynchos ENSAPLP00000015850	45 (7.51)
Lepisosteus oculatus ENSLOCP00000016321	47 (7.85)
Canis lupus ENSCAFP00000025610	47 (7.85)
Erinaceus europaeus ENSEEUP00000002256	47 (7.85)
Homo sapiens ENSP00000475670	47 (7.85)
Meleagris gallopavo ENSMGAP00000002634	47 (7.85)
Anolis carolinensis ENSACAP00000017414	48 (8.01)
Pelodiscus sinensis ENSPSIP00000012541	48 (8.01)
Pteropus vampyrus ENSPVAP00000009482	49 (8.18)
Gallus gallus ENSGALP00000001859	49 (8.18)
Danio rerio ENSDARP00000109865	50 (8.35)
Mus musculus ENSMUSP00000052522	50 (8.35)
Xiphophorus maculatus ENSXMAP00000006614	51 (8.51)
Callorhinchus milii 632942857	51 (8.51)
Oreochromis niloticus ENSONIP00000022879	52 (8.68)
Astyanax mexicanus ENSAMXP00000009984	52 (8.68)
Oryzias latipes ENSORLP00000003864	53 (8.85)
Takifugu rubripes ENSTRUP00000009667	55 (9.18)
Latimeria chalumnae ENSLACP00000004268	56 (9.35)
Bos taurus ENSBTAP00000021739	60 (10.02)
Oreochromis niloticus ENSONIP00000010373	62 (10.35)
Chrysemys picta 530646579	71 (11.85)
Ficedula albicollis ENSFALP00000009808	72 (12.02)
Erinaceus europaeus ENSEEUP00000007413	72 (12.02)
Tupaia belangeri ENSTBEP00000013428	81 (13.52)
Tetraodon nigroviridis ENSTNIP00000017101	131 (21.87)
Tupaia belangeri ENSTBEP00000013236	148 (24.71)
Loxodonta africana ENSLAFP00000005536	249 (41.57)
Latimeria chalumnae ENSLACP00000000071	266 (44.41)
Gasterosteus aculeatus ENSGACP00000025600	366 (61.1)
Gadus morhua ENSGMOP00000019166	370 (61.77)
Gasterosteus aculeatus ENSGACP00000025601	432 (72.12)
Gadus morhua ENSGMOP00000019174	438 (73.12)
