# Supplementary material for: Evolutionary history of phosphatidylinositol- 3-kinases: ancestral origin in eukaryotes and complex duplication patterns
Source: BMC Evol Biol. 2015 Oct 19;15:226. doi: 10.1186/s12862-015-0498-7 (PMC4617754; doi:10.1186/s12862-015-0498-7)
Supplement: Additional file 7 — Number of gaps per sequence after site selection for the MIC class II catalytic subunit dataset. Sequences are sorted by increased percentage of gaps. [file 12862_2015_498_MOESM7_ESM.pdf]

Organism name and sequence ID	Number of gaps (percentage)
Chrysemys picta 530644153	0 (0.0)
Danio rerio ENSDARP00000120821	0 (0.0)
Latimeria chalumnae ENSLACP00000019372	0 (0.0)
Anas platyrhynchos ENSAPLP00000010610	0 (0.0)
Meleagris gallopavo ENSMGAP00000006951	0 (0.0)
Gallus gallus ENSGALP00000009863	0 (0.0)
Taeniopygia guttata ENSTGUP00000008892	0 (0.0)
Ficedula albicollis ENSFALP00000000804	0 (0.0)
Pelodiscus sinensis ENSPSIP00000013783	0 (0.0)
Anolis carolinensis ENSACAP00000000044	0 (0.0)
Mus musculus ENSMUSP00000126092	0 (0.0)
Pteropus vampyrus ENSPVAP00000012080	0 (0.0)
Homo sapiens ENSP00000265970	0 (0.0)
Canis lupus ENSCAFP00000036143	0 (0.0)
Loxodonta africana ENSLAFP00000003094	0 (0.0)
Monodelphis domestica ENSMODP00000034801	0 (0.0)
Ornithorhynchus anatinus ENSOANP00000012246	0 (0.0)
Callorhinchus milii 632941134	1 (0.09)
Chrysemys picta 530639296	1 (0.09)
Lepisosteus oculatus ENSLOCP00000003495	1 (0.09)
Xenopus tropicalis ENSXETP00000034942	1 (0.09)
Pteropus vampyrus ENSPVAP00000006751	1 (0.09)
Homo sapiens ENSP00000356155	1 (0.09)
Otolemur garnettii ENSOGAP00000010659	1 (0.09)
Mus musculus ENSMUSP00000076911	1 (0.09)
Anolis carolinensis ENSACAP00000007419	1 (0.09)
Meleagris gallopavo ENSMGAP00000002505	1 (0.09)
Gallus gallus ENSGALP00000000882	1 (0.09)
Monodelphis domestica ENSMODP00000001932	1 (0.09)
Xiphophorus maculatus ENSXMAP00000012426	2 (0.18)
Oreochromis niloticus ENSONIP00000019580	2 (0.18)
Bos taurus ENSBTAP00000017388	2 (0.18)
Loxodonta africana ENSLAFP00000014283	2 (0.18)
Canis lupus ENSCAFP00000014201	2 (0.18)
Astyanax mexicanus ENSAMXP00000025863	3 (0.27)
Gadus morhua ENSGMOP00000011250	3 (0.27)
Takifugu rubripes ENSTRUP00000038296	4 (0.36)
Tetraodon nigroviridis ENSTNIP00000005672	4 (0.36)
Gasterosteus aculeatus ENSGACP00000008939	4 (0.36)
Latimeria chalumnae ENSLACP00000019798	4 (0.36)
Oryzias latipes ENSORLP00000013515	7 (0.63)
Erinaceus europaeus ENSEEUP00000006595	7 (0.63)
Xiphophorus maculatus ENSXMAP00000012596	9 (0.81)
Apis mellifera 571502742	10 (0.9)
Lepisosteus oculatus ENSLOCP00000014902	10 (0.9)
Danio rerio ENSDARP00000110942	10 (0.9)
Astyanax mexicanus ENSAMXP00000010380	12 (1.08)
Oreochromis niloticus ENSONIP00000000365	14 (1.26)
Drosophila melanogaster FBpp0075818	14 (1.26)
Ficedula albicollis ENSFALP00000000443	15 (1.35)
Tupaia belangeri ENSTBEP00000006933	19 (1.71)
Callorhinchus milii 632963288	27 (2.43)
Gallus gallus ENSGALP00000021380	37 (3.32)
Mus musculus AAI50814	37 (3.32)

Organism name and sequence ID	Number of gaps (percentage)
Loxodonta africana ENSLAFP00000011952	37 (3.32)
Canis lupus ENSCAFP00000018530	37 (3.32)
Bos taurus ENSBTAP00000040697	38 (3.41)
Caenorhabditis elegans F39B1.1	39 (3.5)
Amphimedon queenslandica 340378016	44 (3.95)
Echinococcus granulosus 556520602	49 (4.4)
Erinaceus europaeus ENSEEUP00000013771	56 (5.03)
Pelodiscus sinensis XP006133177	59 (5.3)
Nematostella vectensis 156225157	60 (5.39)
Otolemur garnettii ENSOGAP00000001905	65 (5.84)
Oreochromis niloticus ENSONIP00000010680	68 (6.11)
Monosiga brevicollis 167533638	70 (6.29)
Clonorchis sinensis 358342191	73 (6.56)
Homo sapiens ENSP00000266497	75 (6.74)
Otolemur garnettii ENSOGAP00000002350	77 (6.92)
Schistosoma mansoni 353229343	78 (7.01)
Erinaceus europaeus ENSEEUP00000009401	78 (7.01)
Aplysia californica 524911800	78 (7.01)
Branchiostoma floridae 260790325	79 (7.1)
Pteropus vampyrus ENSPVAP00000000598	79 (7.1)
Latimeria chalumnae ENSLACP00000004519	82 (7.37)
Salpingoeca rosetta 326432665	85 (7.64)
Tupaia belangeri ENSTBEP00000003810	86 (7.73)
Tupaia belangeri ENSTBEP00000011511	86 (7.73)
Xiphophorus maculatus ENSXMAP00000007238	89 (8.0)
Chrysemys picta 530644775	91 (8.18)
Anas platyrhynchos ENSAPLP00000009675	91 (8.18)
Takifugu rubripes ENSTRUP00000043606	96 (8.63)
Capsaspora owczarzaki 470291293	103 (9.25)
Tetraodon nigroviridis ENSTNIP00000008764	109 (9.79)
Ornithorhynchus anatinus XP001506878	116 (10.42)
Taeniopygia guttata ENSTGUP00000012743	129 (11.59)
Meleagris gallopavo ENSMGAP00000014352	141 (12.67)
Ciona intestinalis ENSCINP00000032884	169 (15.18)
Pelodiscus sinensis ENSPSIP00000002245	253 (22.73)
Crassostrea gigas 405975165	268 (24.08)
Petromyzon marinus ENSPMAT00000006926	303 (27.22)
Capitella teleta 443696965	379 (34.05)
Lottia gigantea 556098278	452 (40.61)
Hydra vulgaris 449666338	471 (42.32)
Saccoglossus kowalevskii 585646586	496 (44.56)
Crassostrea gigas 405976593	556 (49.96)
Petromyzon marinus ENSPMAT00000010071	695 (62.44)
Saccoglossus kowalevskii 585718267	716 (64.33)
Bos taurus ENSBTAP00000043907	752 (67.57)
Tetraodon nigroviridis ENSTNIP00000019124	827 (74.3)
Helobdella robusta 555694902	829 (74.48)
Gasterosteus aculeatus ENSGACP00000004463	905 (81.31)
Petromyzon marinus ENSPMAT00000007051	907 (81.49)
Gadus morhua ENSGMOP00000012312	914 (82.12)
Gadus morhua ENSGMOP00000005503	948 (85.18)
Gasterosteus aculeatus ENSGACP00000013210	948 (85.18)
Gasterosteus aculeatus ENSGACP00000004467	969 (87.06)
Gadus morhua ENSGMOP00000012319	969 (87.06)
