# Supplementary material for: Evolutionary history of phosphatidylinositol- 3-kinases: ancestral origin in eukaryotes and complex duplication patterns
Source: BMC Evol Biol. 2015 Oct 19;15:226. doi: 10.1186/s12862-015-0498-7 (PMC4617754; doi:10.1186/s12862-015-0498-7)
Supplement: Additional file 8 — Number of gaps per sequence after site selection for the MIC class I catalytic subunit dataset. Sequences are sorted by increased percentage of gaps. [file 12862_2015_498_MOESM8_ESM.pdf]

Organism name and sequence ID	Number of gaps (percentage)
Nematostella vectensis 156221153	0 (0.0)
Capitella teleta 443734586	1 (0.12)
Callorhinchus milii 632934759	2 (0.24)
Lepisosteus oculatus ENSLOCP00000005286	2 (0.24)
Xiphophorus maculatus ENSXMAP00000006655	2 (0.24)
Oreochromis niloticus ENSONIP00000009986	2 (0.24)
Takifugu rubripes ENSTRUP00000017655	2 (0.24)
Homo sapiens ENSP00000418143	3 (0.36)
Loxodonta africana ENSLAFP00000013155	3 (0.36)
Mus musculus ENSMUSP00000035037	3 (0.36)
Erinaceus europaeus ENSEEUP00000007636	3 (0.36)
Pteropus vampyrus ENSPVAP00000005473	3 (0.36)
Bos taurus ENSBTAP00000009083	3 (0.36)
Canis lupus ENSCAFP00000011245	3 (0.36)
Anolis carolinensis ENSACAP00000003172	3 (0.36)
Pelodiscus sinensis ENSPSIP00000002511	3 (0.36)
Ficedula albicollis ENSFALP00000004251	3 (0.36)
Gallus gallus ENSGALP00000008740	3 (0.36)
Meleagris gallopavo ENSMGAP00000004152	3 (0.36)
Xenopus tropicalis ENSXETP00000056285	3 (0.36)
Latimeria chalumnae ENSLACP00000014968	3 (0.36)
Gadus morhua ENSGMOP00000014980	3 (0.36)
Oryzias latipes ENSORLP00000019450	3 (0.36)
Drosophila melanogaster FBpp0083348	3 (0.36)
Branchiostoma floridae 260815076	4 (0.48)
Taeniopygia guttata ENSTGUP00000005489	4 (0.48)
Danio rerio ENSDARP00000102724	4 (0.48)
Tetraodon nigroviridis ENSTNIP00000017338	4 (0.48)
Lepisosteus oculatus ENSLOCP00000001301	5 (0.6)
Gasterosteus aculeatus ENSGACP00000021206	5 (0.6)
Takifugu rubripes ENSTRUP00000016050	5 (0.6)
Tetraodon nigroviridis ENSTNIP00000010637	5 (0.6)
Oryzias latipes ENSORLP00000011212	5 (0.6)
Oreochromis niloticus ENSONIP00000013444	5 (0.6)
Xiphophorus maculatus ENSXMAP00000004675	5 (0.6)
Gadus morhua ENSGMOP00000003184	5 (0.6)
Anolis carolinensis ENSACAP00000004867	5 (0.6)
Gallus gallus ENSGALP00000014504	5 (0.6)
Meleagris gallopavo ENSMGAP00000010212	5 (0.6)
Anas platyrhynchos ENSAPLP00000012778	5 (0.6)
Ficedula albicollis ENSFALP00000008750	5 (0.6)
Taeniopygia guttata ENSTGUP00000011093	5 (0.6)
Monodelphis domestica ENSMODP00000026633	5 (0.6)
Canis lupus ENSCAFP00000016537	5 (0.6)
Bos taurus ENSBTAP00000012168	5 (0.6)
Otolemur garnettii ENSOGAP00000005899	5 (0.6)
Loxodonta africana ENSLAFP00000002561	5 (0.6)
Homo sapiens ENSP00000263967	5 (0.6)
Danio rerio ENSDARP00000124781	5 (0.6)
Astyanax mexicanus ENSAMXP00000020452	5 (0.6)
Gadus morhua ENSGMOP00000011408	5 (0.6)
Oreochromis niloticus ENSONIP00000022585	5 (0.6)
Xiphophorus maculatus ENSXMAP00000008625	5 (0.6)
Gasterosteus aculeatus ENSGACP00000001555	5 (0.6)
Callorhinchus milii 632968054	5 (0.6)
Callorhinchus milii 632934984	5 (0.6)
Chrysemys picta 530584406	5 (0.6)
Monodelphis domestica ENSMODP00000023181	5 (0.6)
Otolemur garnettii ENSOGAP00000013693	5 (0.6)
Canis lupus ENSCAFP00000029210	5 (0.6)
Mus musculus ENSMUSP00000036434	5 (0.6)
Anas platyrhynchos ENSAPLP00000007963	5 (0.6)
Ficedula albicollis ENSFALP00000007638	5 (0.6)
Gallus gallus ENSGALP00000004061	5 (0.6)
Meleagris gallopavo ENSMGAP00000003869	5 (0.6)
Anolis carolinensis ENSACAP00000013119	5 (0.6)
Pelodiscus sinensis ENSPSIP00000004971	5 (0.6)
Gadus morhua ENSGMOP00000000741	5 (0.6)
Xiphophorus maculatus ENSXMAP00000011635	5 (0.6)
Takifugu rubripes ENSTRUP00000025672	5 (0.6)
Oryzias latipes ENSORLP00000006834	5 (0.6)
Oreochromis niloticus ENSONIP00000002983	5 (0.6)
Mus musculus ENSMUSP00000103878	5 (0.6)
Latimeria chalumnae ENSLACP00000017606	5 (0.6)
Gasterosteus aculeatus ENSGACP00000008159	5 (0.6)
Oreochromis niloticus ENSONIP00000010535	5 (0.6)
Tetraodon nigroviridis ENSTNIP00000009567	5 (0.6)
Takifugu rubripes ENSTRUP00000009530	5 (0.6)
Oryzias latipes ENSORLP00000014606	5 (0.6)
Xiphophorus maculatus ENSXMAP00000000601	5 (0.6)
Lottia gigantea 556102838	6 (0.72)
Crassostrea gigas 405975190	6 (0.72)
Tetraodon nigroviridis ENSTNIP00000018220	6 (0.72)
Gasterosteus aculeatus ENSGACP00000009701	6 (0.72)
Xenopus tropicalis ENSXETP00000015756	7 (0.85)
Anas platyrhynchos ENSAPLP00000003138	7 (0.85)
Gasterosteus aculeatus ENSGACP00000007464	7 (0.85)
Danio rerio ENSDARP00000017757	7 (0.85)
Lepisosteus oculatus ENSLOCP00000008374	7 (0.85)
Capitella teleta 443701283	8 (0.97)
Tupaia belangeri ENSTBEP00000010880	9 (1.09)
Tetraodon nigroviridis ENSTNIP00000005428	9 (1.09)
Takifugu rubripes ENSTRUP00000013971	10 (1.21)

Organism name and sequence ID	Number of gaps (percentage)
Ciona savignyi ENSCSAVP00000004359	11 (1.33)
Caenorhabditis elegans B0334.8	12 (1.45)
Schistosoma mansoni 353228720	13 (1.57)
Trichoplax adhaerens 196007024	13 (1.57)
Clonorchis sinensis 358334260	13 (1.57)
Xenopus tropicalis ENSXETP00000013019	16 (1.93)
Oryzias latipes ENSORLP00000011853	18 (2.17)
Xiphophorus maculatus ENSXMAP00000008835	18 (2.17)
Latimeria chalumnae ENSLACP00000007893	18 (2.17)
Gallus gallus ENSGALP00000013101	18 (2.17)
Meleagris gallopavo ENSMGAP00000014459	18 (2.17)
Taeniopygia guttata ENSTGUP00000003158	18 (2.17)
Anas platyrhynchos ENSAPLP00000006296	18 (2.17)
Pelodiscus sinensis ENSPSIP00000006579	18 (2.17)
Branchiostoma floridae 260831840	18 (2.17)
Lepisosteus oculatus ENSLOCP00000019486	19 (2.29)
Oreochromis niloticus ENSONIP00000014923	19 (2.29)
Helobdella robusta 555689542	19 (2.29)
Xiphophorus maculatus ENSXMAP00000016592	19 (2.29)
Apis mellifera 571563007	19 (2.29)
Ciona intestinalis ENSCINP00000010684	19 (2.29)
Gasterosteus aculeatus ENSGACP00000025368	20 (2.42)
Tetraodon nigroviridis ENSTNIP00000009686	20 (2.42)
Loxodonta africana ENSLAFP00000003840	20 (2.42)
Otolemur garnettii ENSOGAP00000011733	20 (2.42)
Homo sapiens ENSP00000392258	20 (2.42)
Canis lupus ENSCAFP00000005911	20 (2.42)
Mus musculus ENSMUSP00000082596	20 (2.42)
Anolis carolinensis ENSACAP00000014629	20 (2.42)
Callorhinchus milii 632943520	20 (2.42)
Amphimedon queenslandica 340370959	20 (2.42)
Tupaia belangeri ENSTBEP00000008487	21 (2.54)
Callorhinchus milii 632946473	21 (2.54)
Capsaspora owczarzaki 470309276	21 (2.54)
Astyanax mexicanus ENSAMXP00000004300	22 (2.66)
Danio rerio ENSDARP00000002818	22 (2.66)
Monodelphis domestica ENSMODP00000020477	22 (2.66)
Oreochromis niloticus ENSONIP00000002013	22 (2.66)
Helobdella robusta 555699397	22 (2.66)
Ciona intestinalis ENSCINP00000014231	23 (2.78)
Latimeria chalumnae ENSLACP00000012857	23 (2.78)
Homo sapiens ENSP00000446444	23 (2.78)
Bos taurus ENSBTAP00000025274	23 (2.78)
Pteropus vampyrus ENSPVAP00000004422	24 (2.9)
Amphimedon queenslandica 340370156	25 (3.02)
Ornithorhynchus anatinus ENSOANP00000012983	25 (3.02)
Gadus morhua ENSGMOP00000009961	26 (3.14)
Lepisosteus oculatus ENSLOCP00000016225	26 (3.14)
Capsaspora owczarzaki 470303443	26 (3.14)
Monosiga brevicollis 167521039	26 (3.14)
Petromyzon marinus ENSPMAT00000002453	31 (3.74)
Echinococcus granulosus 576697681	31 (3.74)
Lottia gigantea 556100131	33 (3.99)
Danio rerio ENSDARP00000054650	34 (4.11)
Oryzias latipes ENSORLP00000002186	34 (4.11)
Ciona savignyi ENSCSAVP00000000553	36 (4.35)
Pteropus vampyrus ENSPVAP00000013295	37 (4.47)
Latimeria chalumnae ENSLACP00000010097	37 (4.47)
Ornithorhynchus anatinus ENSOANP00000023160	41 (4.95)
Pelodiscus sinensis ENSPSIP00000016189	43 (5.19)
Loxodonta africana XP003413227	46 (5.56)
Gasterosteus aculeatus ENSGACP00000005515	53 (6.4)
Takifugu rubripes ENSTRUP00000008194	54 (6.52)
Tetraodon nigroviridis ENSTNIP00000003526	55 (6.64)
Amphimedon queenslandica 340369052	56 (6.76)
Gadus morhua ENSGMOP00000000052	58 (7.0)
Aplysia californica 524908046	65 (7.85)
Ciona savignyi ENSCSAVP00000007755	68 (8.21)
Erinaceus europaeus ENSEEUP00000013678	71 (8.57)
Takifugu rubripes XP003973030	73 (8.82)
Tupaia belangeri ENSTBEP00000005289	78 (9.42)
Salpingoeca rosetta 326430078	79 (9.54)
Aplysia californica 524899805	92 (11.11)
Tupaia belangeri ENSTBEP00000002551	93 (11.23)
Pteropus vampyrus ENSPVAP00000002313	98 (11.84)
Erinaceus europaeus ENSEEUP00000006927	104 (12.56)
Crassostrea gigas 405975626	109 (13.16)
Ficedula albicollis ENSFALP00000010709	129 (15.58)
Tetraodon nigroviridis ENSTNIP00000006420	148 (17.87)
Saccoglossus kowalevskii 585654121	150 (18.12)
Otolemur garnettii ENSOGAP00000003608	152 (18.36)
Salpingoeca rosetta 326435786	159 (19.2)
Monodelphis domestica ENSMODP00000003651	171 (20.65)
Bos taurus ENSBTAP00000027780	219 (26.45)
Hydra vulgaris 449686903	421 (50.85)
Hydra vulgaris 449686315	437 (52.78)
Petromyzon marinus ENSPMAT00000002863	438 (52.9)
Saccoglossus kowalevskii 585661864	459 (55.43)
Amphimedon queenslandica 340383255	486 (58.7)
Petromyzon marinus ENSPMAT00000000829	573 (69.2)
Monosiga brevicollis 167520402	599 (72.34)
Saccoglossus kowalevskii 585706768	599 (72.34)
