# Supplementary figures and images for: Evolutionary history of phosphatidylinositol- 3-kinases: ancestral origin in eukaryotes and complex duplication patterns
Source: BMC Evol Biol. 2015 Oct 19;15:226. doi: 10.1186/s12862-015-0498-7 (PMC4617754; doi:10.1186/s12862-015-0498-7)

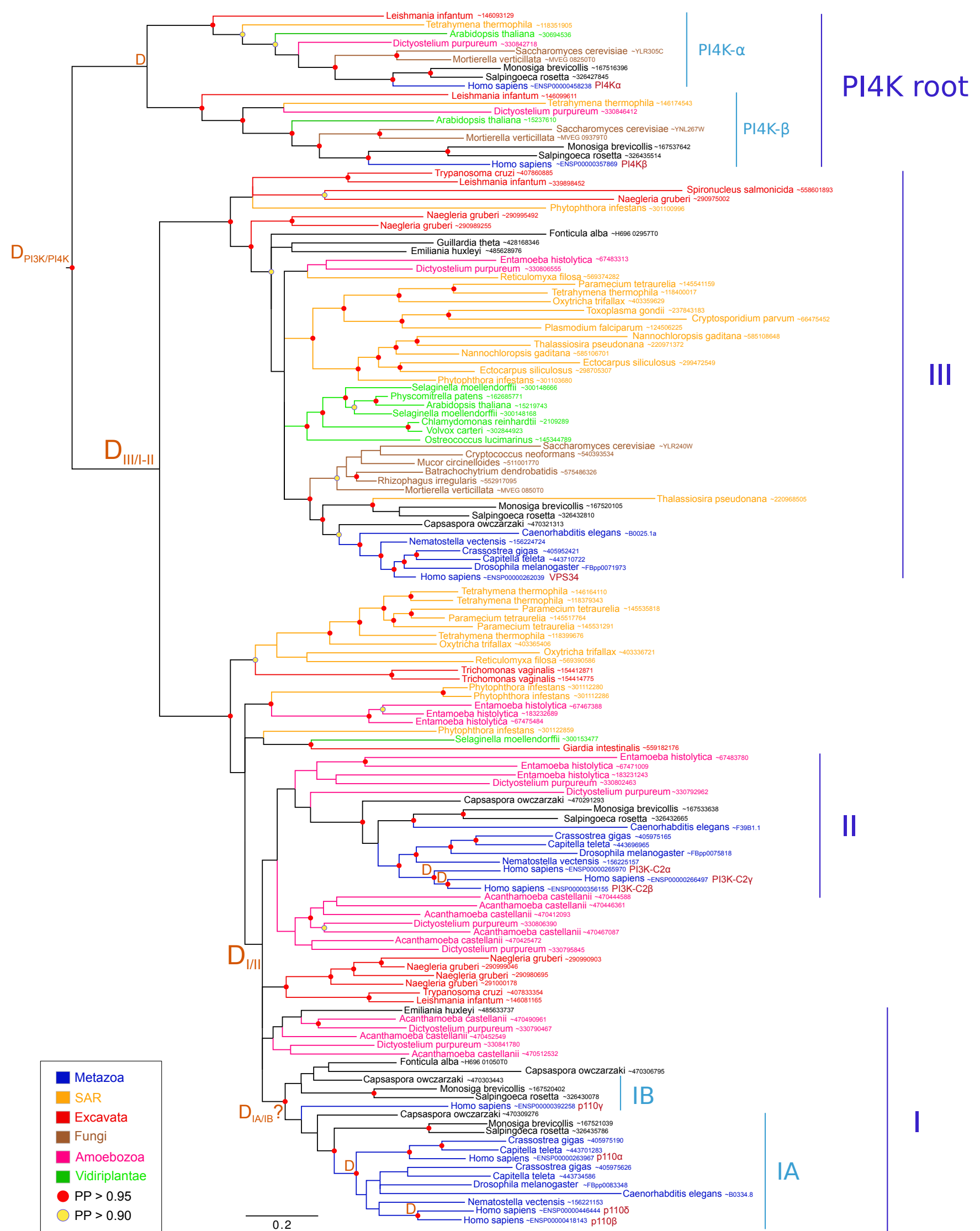

Supplement: Additional file 10 — Bayesian phylogenetic tree of selected catalytic subunits. The tree was inferred using the MrBayes program and the same alignment as the one used to build the corresponding maximum likelihood tree (Fig. 1). Sequences are colored according to their taxonomic classification. Yellow and red circles correspond to PP >0.90 and PP >0.95, respectively. Duplication events are indicated by an orange “D”. The scale bar represents the average number of substitutions per site. [file 12862_2015_498_MOESM10_ESM.pdf]
