# Supplementary material for: Evolutionary history of phosphatidylinositol- 3-kinases: ancestral origin in eukaryotes and complex duplication patterns
Source: BMC Evol Biol. 2015 Oct 19;15:226. doi: 10.1186/s12862-015-0498-7 (PMC4617754; doi:10.1186/s12862-015-0498-7)
Supplement: Additional file 9 — Complete phylogenetic tree of catalytic subunits. The tree was inferred with the JTT+ Γ 4 model (468 sites, 1055 sequences). Sequences are colored according to their taxonomic classification. SH support is indicated over the branches. Duplication events are shown by an orange“D”. The scale bar represents the average number of substitutions per site. [file 12862_2015_498_MOESM9_ESM.pdf]

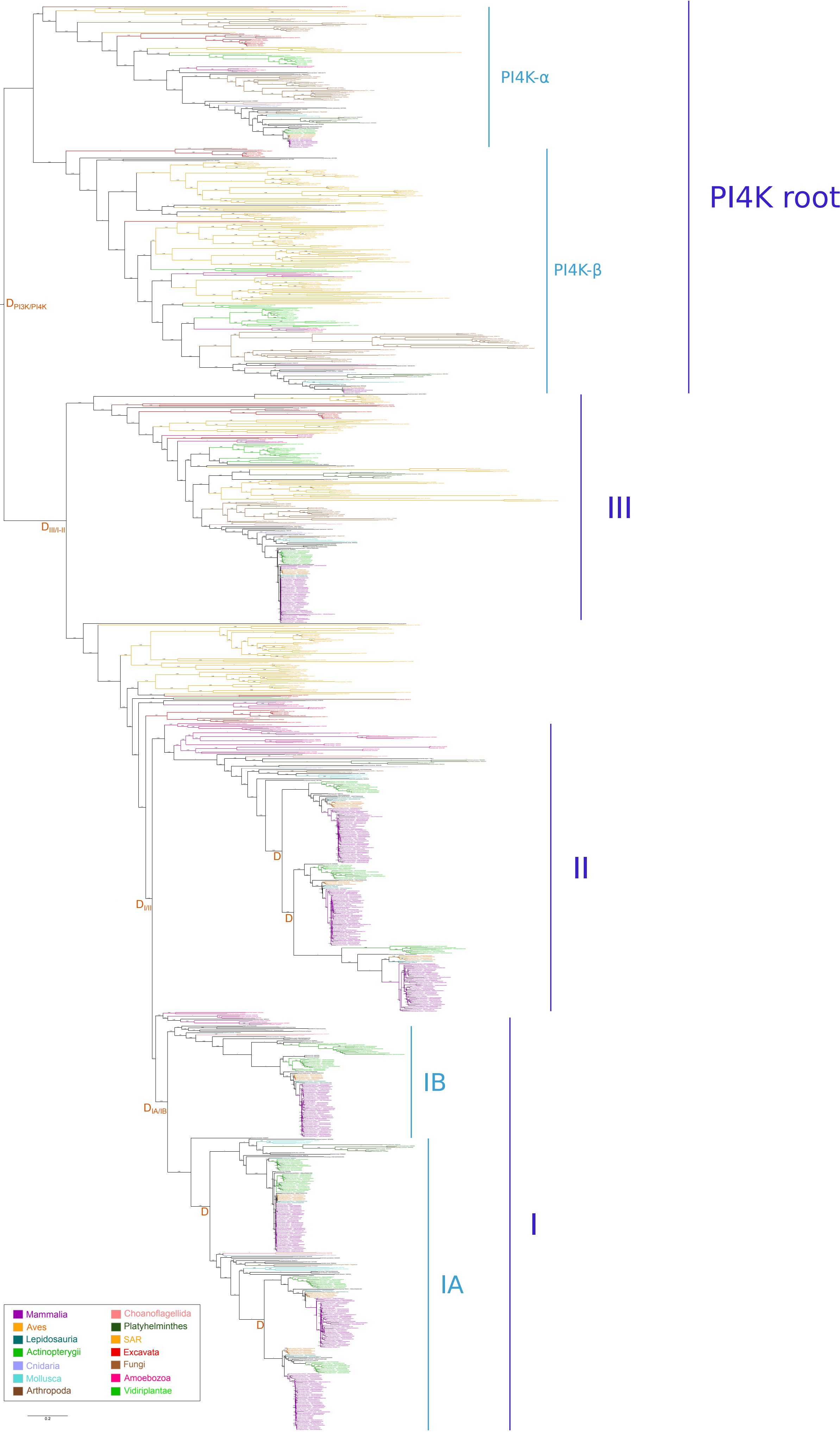
PI4K-α
PI4K-β
PI4K root
$D_{PI3K/PI4K}$
$D_{III/I-II}$
III
II
$D_{I/II}$
D
D
IB
$D_{IA/IB}$
D
I
IA
D
Mammalia
Aves
Lepidosauria
Actinopterygii
Cnidaria
Mollusca
Arthropoda
Choanoflagellida
Platyhelminthes
SAR
Excavata
Fungi
Amoebozoa
Vidiriplantae
0.2
