# Supplementary material for: Evolutionary history of phosphatidylinositol- 3-kinases: ancestral origin in eukaryotes and complex duplication patterns
Source: BMC Evol Biol. 2015 Oct 19;15:226. doi: 10.1186/s12862-015-0498-7 (PMC4617754; doi:10.1186/s12862-015-0498-7)
Supplement: Additional file 11 — Phylogenetic tree of Metazoa, Ichthyosporea and Choanoflagellida homologs of class II catalytic proteins. The tree was inferred with the JTT+ Γ 4 model (1113 sites, 108 sequences). Sequences are colored according to their taxonomic classification. Branch statistical supports and duplication events are shown using the same symbols as in Fig. 1. As described in the Material and methods section, we selected all non-mammal species from Ensembl, and kept ten representative mammal organisms and all Ichtyosporea, Choanoflagellida and Metazoa species from our local database. [file 12862_2015_498_MOESM11_ESM.pdf]

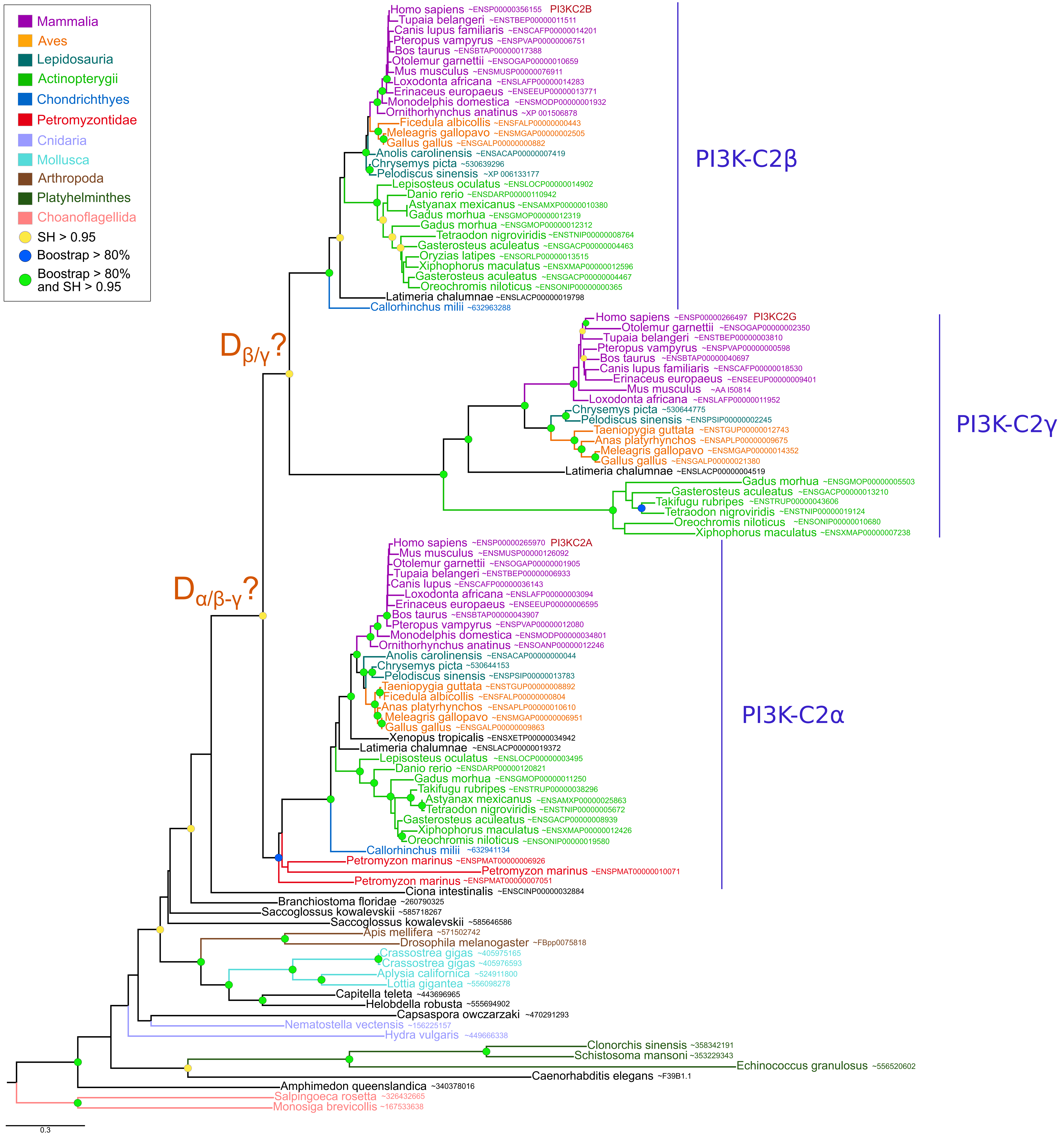

Mammalia
Aves
Lepidosauria
Actinopterygii
Chondrichthyes
Petromyzontidae
Cnidaria
Mollusca
Arthropoda
Platyhelminthes
Choanoflagellida
SH > 0.95
Boostrap > 80%
Boostrap > 80% and SH > 0.95
Homo sapiens ~ENSP00000356155 PI3KC2B
Tupaia belangeri ~ENSTBEP00000011511
Canis lupus familiaris ~ENSCAFP00000014201
Pteropus vampyrus ~ENSPVAP00000006751
Bos taurus ~ENSBTAP00000017388
Otolemur garnettii ~ENSOGAP00000010659
Mus musculus ~ENSMUSP00000076911
Loxodonta africana ~ENSLAFP00000014283
Erinaceus europaeus ~ENSEEUP00000013771
Monodelphis domestica ~ENSMODP00000001932
Ornithorhynchus anatinus ~XP 001506878
Ficedula albicollis ~ENSFALP00000000443
Meleagris gallopavo ~ENSMGAP00000002505
Gallus gallus ~ENSGALP00000000882
Anolis carolinensis ~ENSACAP00000007419
Chrysemys picta ~530639296
Pelodiscus sinensis ~XP 006133177
Lepisosteus oculatus ~ENSLOCP00000014902
Danio rerio ~ENSDARP00000110942
Astyanax mexicanus ~ENSAMXP00000010380
Gadus morhua ~ENSGMOP00000012319
Gadus morhua ~ENSGMOP00000012312
Tetraodon nigroviridis ~ENSTNIP00000008764
Gasterosteus aculeatus ~ENSGACP00000004463
Oryzias latipes ~ENSORLP00000013515
Xiphophorus maculatus ~ENSXMAP00000012596
Gasterosteus aculeatus ~ENSGACP00000004467
Oreochromis niloticus ~ENSONIP00000000365
Latimeria chalumnae ~ENSLACP00000019798
Callorhinchus milii ~632963288
PI3K-C2β
Dβ/γ?
Homo sapiens ~ENSP00000266497 PI3KC2G
Otolemur garnettii ~ENSOGAP00000002350
Tupaia belangeri ~ENSTBEP00000003810
Pteropus vampyrus ~ENSPVAP00000000598
Bos taurus ~ENSBTAP00000040697
Canis lupus familiaris ~ENSCAFP00000018530
Erinaceus europaeus ~ENSEEUP00000009401
Mus musculus ~AA I50814
Loxodonta africana ~ENSLAFP00000011952
Chrysemys picta ~530644775
Pelodiscus sinensis ~ENSPSIP00000002245
Taeniopygia guttata ~ENSTGUP00000012743
Anas platyrhynchos ~ENSAPLP00000009675
Meleagris gallopavo ~ENSMGAP00000014352
Gallus gallus ~ENSGALP00000021380
Latimeria chalumnae ~ENSLACP00000004519
Gadus morhua ~ENSGMOP00000005503
Gasterosteus aculeatus ~ENSGACP00000013210
Takifugu rubripes ~ENSTRUP00000043606
Tetraodon nigroviridis ~ENSTNIP00000019124
Oreochromis niloticus ~ENSONIP00000010680
Xiphophorus maculatus ~ENSXMAP00000007238
PI3K-C2γ
Dα/β-γ?
Homo sapiens ~ENSP00000265970 PI3KC2A
Mus musculus ~ENSMUSP00000126092
Otolemur garnettii ~ENSOGAP00000001905
Tupaia belangeri ~ENSTBEP00000006933
Canis lupus ~ENSCAFP00000036143
Loxodonta africana ~ENSLAFP00000003094
Erinaceus europaeus ~ENSEEUP00000006595
Bos taurus ~ENSBTAP00000043907
Pteropus vampyrus ~ENSPVAP00000012080
Monodelphis domestica ~ENSMODP00000034801
Ornithorhynchus anatinus ~ENSOANP00000012246
Anolis carolinensis ~ENSACAP00000000044
Chrysemys picta ~530644153
Pelodiscus sinensis ~ENSPSIP00000013783
Taeniopygia guttata ~ENSTGUP00000008892
Ficedula albicollis ~ENSFALP00000000804
Anas platyrhynchos ~ENSAPLP00000010610
Meleagris gallopavo ~ENSMGAP00000006951
Gallus gallus ~ENSGALP00000009863
Xenopus tropicalis ~ENSXETP00000034942
Latimeria chalumnae ~ENSLACP00000019372
Lepisosteus oculatus ~ENSLOCP00000003495
Danio rerio ~ENSDARP00000120821
Gadus morhua ~ENSGMOP00000011250
Takifugu rubripes ~ENSTRUP00000038296
Astyanax mexicanus ~ENSAMXP00000025863
Tetraodon nigroviridis ~ENSTNIP00000005672
Gasterosteus aculeatus ~ENSGACP00000008939
Xiphophorus maculatus ~ENSXMAP00000012426
Oreochromis niloticus ~ENSONIP00000019580
Callorhinchus milii ~632941134
Petromyzon marinus ~ENSPMAT00000006926
Petromyzon marinus ~ENSPMAT00000010071
Petromyzon marinus ~ENSPMAT00000007051
PI3K-C2α
Ciona intestinalis ~ENSCINP00000032884
Branchiostoma floridae ~260790325
Saccoglossus kowalevskii ~585718267
Saccoglossus kowalevskii ~585646586
Apis mellifera ~571502742
Drosophila melanogaster ~FBpp0075818
Crassostrea gigas ~405975165
Crassostrea gigas ~405976593
Aplysia californica ~524911800
Lottia gigantea ~556098278
Capitella teleta ~443696965
Helobdella robusta ~555694902
Capsaspora owczarzaki ~470291293
Nematostella vectensis ~156225157
Hydra vulgaris ~449666338
Clonorchis sinensis ~358342191
Schistosoma mansoni ~353229343
Echinococcus granulosus ~556520602
Caenorhabditis elegans ~F39B1.1
Amphimedon queenslandica ~340378016
Salpingoeca rosetta ~326432665
Monosiga brevicollis ~167533638
0.3
