# Supplementary material for: Evolutionary history of phosphatidylinositol- 3-kinases: ancestral origin in eukaryotes and complex duplication patterns
Source: BMC Evol Biol. 2015 Oct 19;15:226. doi: 10.1186/s12862-015-0498-7 (PMC4617754; doi:10.1186/s12862-015-0498-7)
Supplement: Additional file 12 — Phylogenetic tree of Metazoa, Ichtyosporea and Choanoflagellida homologs of PI3K class I catalytic proteins. The tree was inferred with the LG+ Γ 4 model (828 sites, 185 sequences). Sequences are colored according to their taxonomic classification. Branch statistical supports and duplication events are shown using the same symbols as in Fig. 1. [file 12862_2015_498_MOESM12_ESM.pdf]

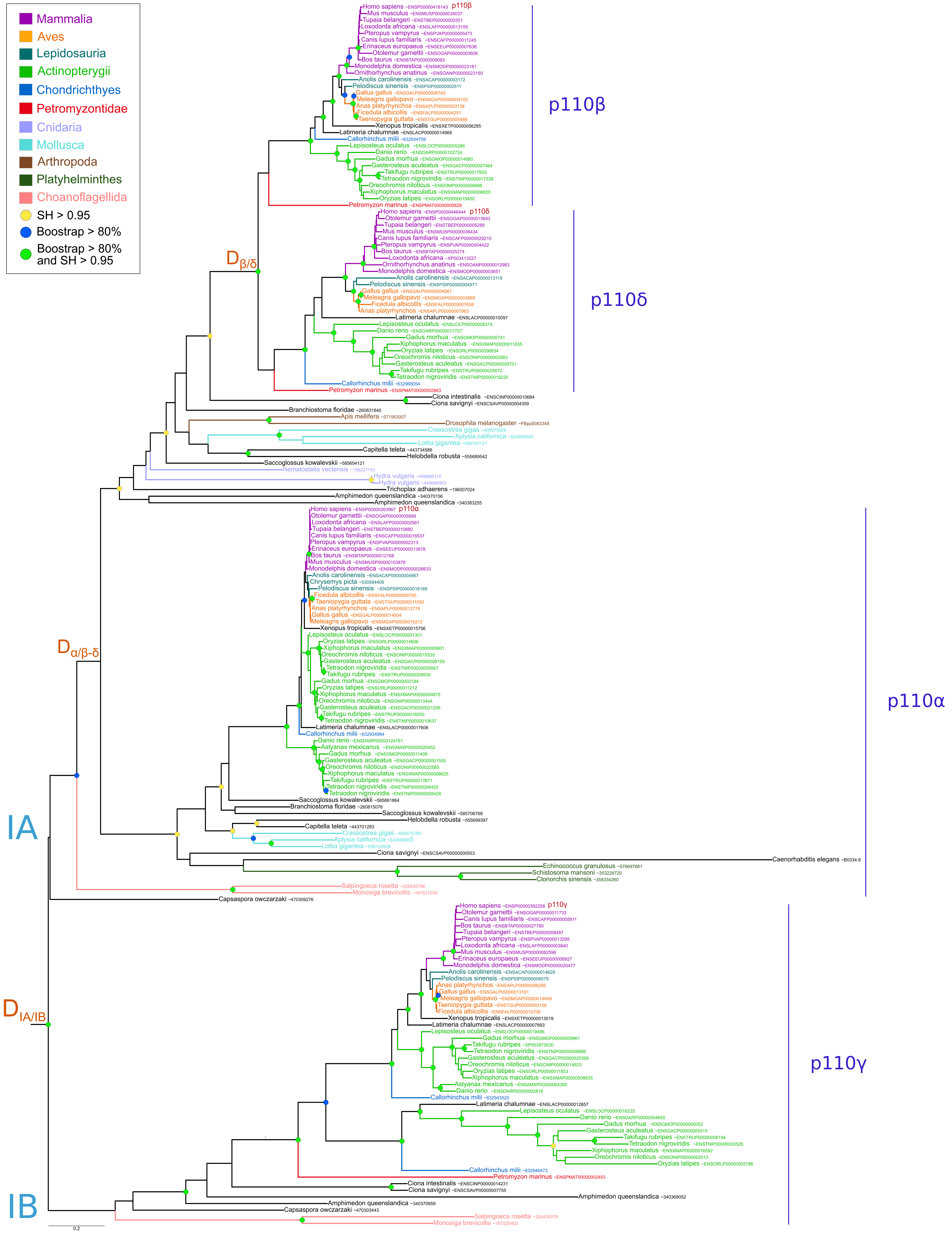

Mammalia
Aves
Lepidosauria
Actinopterygii
Chondrichthyes
Petromyzontidae
Cnidaria
Mollusca
Arthropoda
Platyhelminthes
Choanoflagellida
SH > 0.95
Boostrap > 80%
Boostrap > 80% and SH > 0.95
Homo sapiens ~ENSP00000418143 p110β
Mus musculus ~ENSMUSP00000035037
Tupaia belangeri ~ENSTBEP00000002551
Loxodonta africana ~ENSLAFP00000013155
Pteropus vampyrus ~ENSPVAP00000005473
Canis lupus familiaris ~ENSCAFP00000011245
Erinaceus europaeus ~ENSEEUP00000007636
Otolemur garnettii ~ENSOGAP00000003608
Bos taurus ~ENSBTAP00000009083
Monodelphis domestica ~ENSMODP00000023181
Ornithorhynchus anatinus ~ENSOANP00000023160
Anolis carolinensis ~ENSACAP00000003172
Pelodiscus sinensis ~ENSPSIP00000002511
Gallus gallus ~ENSGALP00000008740
Meleagris gallopavo ~ENSMGAP00000004152
Anas platyrhynchos ~ENSAPLP00000003138
Ficedula albicollis ~ENSFALP00000004251
Taeniopygia guttata ~ENSTGUP00000005489
Xenopus tropicalis ~ENSXETP00000056285
Latimeria chalumnae ~ENSLACP00000014968
Callorhinchus milii ~632934759
Lepisosteus oculatus ~ENSLOCP00000005286
Danio rerio ~ENSDARP00000102724
Gadus morhua ~ENSGMOP00000014980
Gasterosteus aculeatus ~ENSGACP00000007464
Takifugu rubripes ~ENSTRUP00000017655
Tetraodon nigroviridis ~ENSTNIP00000017338
Oreochromis niloticus ~ENSONIP00000009986
Xiphophorus maculatus ~ENSXMAP00000006655
Oryzias latipes ~ENSORLP00000019450
Petromyzon marinus ~ENSPMAT00000000829
p110β
Homo sapiens ~ENSP00000446444 p110δ
Otolemur garnettii ~ENSOGAP00000013693
Tupaia belangeri ~ENSTBEP00000005289
Mus musculus ~ENSMUSP00000036434
Canis lupus familiaris ~ENSCAFP00000029210
Pteropus vampyrus ~ENSPVAP00000004422
Bos taurus ~ENSBTAP00000025274
Loxodonta africana ~XP003413227
Ornithorhynchus anatinus ~ENSOANP00000012983
Monodelphis domestica ~ENSMODP00000003651
Anolis carolinensis ~ENSACAP00000013119
Pelodiscus sinensis ~ENSPSIP00000004971
Gallus gallus ~ENSGALP00000004061
Meleagris gallopavo ~ENSMGAP00000003869
Ficedula albicollis ~ENSFALP00000007638
Anas platyrhynchos ~ENSAPLP00000007963
Latimeria chalumnae ~ENSLACP00000010097
Lepisosteus oculatus ~ENSLOCP00000008374
Danio rerio ~ENSDARP00000017757
Gadus morhua ~ENSGMOP00000000741
Xiphophorus maculatus ~ENSXMAP00000011635
Oryzias latipes ~ENSORLP00000006834
Oreochromis niloticus ~ENSONIP00000002983
Gasterosteus aculeatus ~ENSGACP00000009701
Takifugu rubripes ~ENSTRUP00000025672
Tetraodon nigroviridis ~ENSTNIP00000018220
Callorhinchus milii ~632968054
Petromyzon marinus ~ENSPMAT00000002863
p110δ
Dβ/δ
Ciona intestinalis ~ENSCINP00000010684
Ciona savignyi ~ENSCSAVP00000004359
Branchiostoma floridae ~260831840
Apis mellifera ~571563007
Drosophila melanogaster ~FBpp0083348
Crassostrea gigas ~405975626
Aplysia californica ~524908046
Lottia gigantea ~556100131
Capitella teleta ~443734586
Helobdella robusta ~555689542
Saccoglossus kowalevskii ~585654121
Nematostella vectensis ~156221153
Hydra vulgaris ~449686315
Hydra vulgaris ~449686903
Trichoplax adhaerens ~196007024
Amphimedon queenslandica ~340370156
Amphimedon queenslandica ~340383255
Homo sapiens ~ENSP00000263967 p110α
Otolemur garnettii ~ENSOGAP00000005899
Loxodonta africana ~ENSLAFP00000002561
Tupaia belangeri ~ENSTBEP00000010880
Canis lupus familiaris ~ENSCAFP00000016537
Pteropus vampyrus ~ENSPVAP00000002313
Erinaceus europaeus ~ENSEEUP00000013678
Bos taurus ~ENSBTAP00000012168
Mus musculus ~ENSMUSP00000103878
Monodelphis domestica ~ENSMODP00000026633
Anolis carolinensis ~ENSACAP00000004867
Chrysemys picta ~530584406
Pelodiscus sinensis ~ENSPSIP00000016189
Ficedula albicollis ~ENSFALP00000008750
Taeniopygia guttata ~ENSTGUP00000011093
Anas platyrhynchos ~ENSAPLP00000012778
Gallus gallus ~ENSGALP00000014504
Meleagris gallopavo ~ENSMGAP00000010212
Xenopus tropicalis ~ENSXETP00000015756
Lepisosteus oculatus ~ENSLOCP00000001301
Oryzias latipes ~ENSORLP00000014606
Xiphophorus maculatus ~ENSXMAP00000000601
Oreochromis niloticus ~ENSONIP00000010535
Gasterosteus aculeatus ~ENSGACP00000008159
Tetraodon nigroviridis ~ENSTNIP00000009567
Takifugu rubripes ~ENSTRUP00000009530
Gadus morhua ~ENSGMOP00000003184
Oryzias latipes ~ENSORLP00000011212
Xiphophorus maculatus ~ENSXMAP00000004675
Oreochromis niloticus ~ENSONIP00000013444
Gasterosteus aculeatus ~ENSGACP00000021206
Takifugu rubripes ~ENSTRUP00000016050
Tetraodon nigroviridis ~ENSTNIP00000010637
Latimeria chalumnae ~ENSLACP00000017606
Callorhinchus milii ~632934984
Danio rerio ~ENSDARP00000124781
Astyanax mexicanus ~ENSAMXP00000020452
Gadus morhua ~ENSGMOP00000011408
Gasterosteus aculeatus ~ENSGACP00000001555
Oreochromis niloticus ~ENSONIP00000022585
Xiphophorus maculatus ~ENSXMAP00000008625
Takifugu rubripes ~ENSTRUP00000013971
Tetraodon nigroviridis ~ENSTNIP00000006420
Tetraodon nigroviridis ~ENSTNIP00000005428
Saccoglossus kowalevskii ~585661864
Branchiostoma floridae ~260815076
Saccoglossus kowalevskii ~585706768
Helobdella robusta ~555699397
Capitella teleta ~443701283
Crassostrea gigas ~405975190
Aplysia californica ~524899805
Lottia gigantea ~556102838
Ciona savignyi ~ENSCSAVP00000000553
Caenorhabditis elegans ~B0334.8
Echinococcus granulosus ~576697681
Schistosoma mansoni ~353228720
Clonorchis sinensis ~358334260
Salpingoeca rosetta ~326435786
Monosiga brevicollis ~167521039
Capsaspora owczarzaki ~470309276
p110α
Dα/β-δ
IA
Homo sapiens ~ENSP00000392258 p110γ
Otolemur garnettii ~ENSOGAP00000011733
Canis lupus familiaris ~ENSCAFP00000005911
Bos taurus ~ENSBTAP00000027780
Tupaia belangeri ~ENSTBEP00000008487
Pteropus vampyrus ~ENSPVAP00000013295
Loxodonta africana ~ENSLAFP00000003840
Mus musculus ~ENSMUSP00000082596
Erinaceus europaeus ~ENSEEUP00000006927
Monodelphis domestica ~ENSMODP00000020477
Anolis carolinensis ~ENSACAP00000014629
Pelodiscus sinensis ~ENSPSIP00000006579
Anas platyrhynchos ~ENSAPLP00000006296
Gallus gallus ~ENSGALP00000013101
Meleagris gallopavo ~ENSMGAP00000014459
Taeniopygia guttata ~ENSTGUP00000003158
Ficedula albicollis ~ENSFALP00000010709
Xenopus tropicalis ~ENSXETP00000013019
Latimeria chalumnae ~ENSLACP00000007893
Lepisosteus oculatus ~ENSLOCP00000019486
Gadus morhua ~ENSGMOP00000009961
Takifugu rubripes ~XP003973030
Tetraodon nigroviridis ~ENSTNIP00000009686
Gasterosteus aculeatus ~ENSGACP00000025368
Oreochromis niloticus ~ENSONIP00000014923
Oryzias latipes ~ENSORLP00000011853
Xiphophorus maculatus ~ENSXMAP00000008835
Astyanax mexicanus ~ENSAMXP00000004300
Danio rerio ~ENSDARP00000002818
Callorhinchus milii ~632943520
Latimeria chalumnae ~ENSLACP00000012857
Lepisosteus oculatus ~ENSLOCP00000016225
Danio rerio ~ENSDARP00000054650
Gadus morhua ~ENSGMOP00000000052
Gasterosteus aculeatus ~ENSGACP00000005515
Takifugu rubripes ~ENSTRUP00000008194
Tetraodon nigroviridis ~ENSTNIP00000003526
Xiphophorus maculatus ~ENSXMAP00000016592
Oreochromis niloticus ~ENSONIP00000002013
Oryzias latipes ~ENSORLP00000002186
Callorhinchus milii ~632946473
Petromyzon marinus ~ENSPMAT00000002453
Ciona intestinalis ~ENSCINP00000014231
Ciona savignyi ~ENSCSAVP00000007755
Amphimedon queenslandica ~340369052
Amphimedon queenslandica ~340370959
Capsaspora owczarzaki ~470303443
Salpingoeca rosetta ~326430078
Monosiga brevicollis ~167520402
p110γ
DIA/IB
IB
0.2
